# Effect of BI-1 on insulin resistance through regulation of CYP2E1

**DOI:** 10.1038/srep32229

**Published:** 2016-08-31

**Authors:** Geum-Hwa Lee, Kyoung-Jin Oh, Hyung-Ryong Kim, Hye-Sook Han, Hwa-Young Lee, Keun-Gyu Park, Ki-Hoan Nam, Seung-Hoi Koo, Han-Jung Chae

**Affiliations:** 1 Department of Pharmacology and New Drug Development Institute, Medical School, Chonbuk National University, Jeonju, 561-181, Republic of Korea; 2 Division of Life Sciences, Korea University, 145 Anam-Ro, Seongbuk-Gu, Seoul, 136-713, Republic of Korea; 3 Metabolic Regulation Research Center, Korea Research Institute of Bioscience and Biotechnology (KRIBB), Daejeon, 305-806, Republic of Korea; 4 Department of Dental Pharmacology and Wonkwang Dental Research Institute, School of Dentistry, Wonkwang University, Iksan, 570-749, Republic of Korea; 5 Department of Internal Medicine, Kyungpook National University School of Medicine, Daegu, 700-721, Republic of Korea; 6 Laboratory Animal Resource Center, KRIBB, Ochang-eup, 363-883, Republic of Korea

## Abstract

Diet-induced obesity is a major contributing factor to the progression of hepatic insulin resistance. Increased free fatty acids in liver enhances endoplasmic reticulum (ER) stress and production of reactive oxygen species (ROS), both are directly responsible for dysregulation of hepatic insulin signaling. BI-1, a recently studied ER stress regulator, was examined to investigate its association with ER stress and ROS in insulin resistance models. To induce obesity and insulin resistance, BI-1 wild type and BI-1 knock-out mice were fed a high-fat diet for 8 weeks. The BI-1 knock-out mice had hyperglycemia, was associated with impaired glucose and insulin tolerance under high-fat diet conditions. Increased activity of NADPH-dependent CYP reductase-associated cytochrome p450 2E1 (CYP2E1) and exacerbation of ER stress in the livers of BI-1 knock-out mice was also observed. Conversely, stable expression of BI-1 in HepG2 hepatocytes was shown to reduce palmitate-induced ER stress and CYP2E1-dependent ROS production, resulting in the preservation of intact insulin signaling. Stable expression of CYP2E1 led to increased ROS production and dysregulation of insulin signaling in hepatic cells, mimicking palmitate-mediated hepatic insulin resistance. We propose that BI-1 protects against obesity-induced hepatic insulin resistance by regulating CYP2E1 activity and ROS production.

Anti-apoptotic protein Bax inhibitor-1 (BI-1) protects against apoptosis induced by ER stress^1^,^2^,^3^. In previous BI-1 studies, reactive oxygen species (ROS) regulation characteristics have been investigated^2^,^4^. In ER, the major source of ROS is the microsomal monooxygenase system. Although the main function of the microsomal monooxygenase system is to oxygenate exogenous compounds (xenobiotics) and some endogenous substrates, the microsomal monooxygenase system also produces reactive oxygen species (ROS), such as superoxide anion radical and H_2_O_2_^5^,^6^,^7^. The efficiency, or degree of coupling, of electron transfer from NADPH to CYP is usually <50–60%, and is often as low as 0.5–3.0%. This ‘electron leakage’ contributes significantly to ROS production during redox cycling between NADPH-cytochrome CYP reductase (CPR) and eukaryotic CYPs. ROS production is increased by electron leakage from ER stress-associated CYP2E1 activation, which itself is inhibited by BI-1 overexpression^8^.

The inhibitory effect of BI-1 on ROS production has been primarily linked to cell protection mechanisms. Although direct association with the ER stress signaling pathway has not been formally established, an abundance of ROS often coincides with insulin resistance^9^,^10^,^11^. Increased hepatic CYP2E1 protein expression and activity in association with enhanced ROS generation have recently been reported in animal models of obesity and in patients with obesity and both alcoholic and nonalcoholic fatty liver disease^12^,^13^. These results suggest a role for increased ROS generation in the pathogenesis of insulin resistance.

Insulin resistance consists of a cluster of metabolic disorders. Hepatic lipid accumulation is strongly associated with insulin resistance^11^, and it is widely accepted that lipid accumulation represents the hepatic manifestations of systemic impairments in the insulin network. Insulin resistance studies have demonstrated that phosphorylation of IRS1/2 is controlled by JNK (c-Jun N-terminal kinase) phosphorylation as a signal transduction mechanism^14^,^15^,^16^. It has also been suggested that elevated ROS levels impair insulin signaling by way of several complex mechanisms, including phosphorylation of JNK and the subsequent downstream serine/threonine phosphorylation of IRS1^17^,^18^. Elevation of ROS levels has been proposed as a link between free fatty acid and hepatic insulin resistance^19^. In this study, we hypothesized that palmitate-induced ER stress results in the accumulation of ROS, originating from the microsomal monooxygenase system in liver cells. We evaluated the role of BI-1 in the microsomal monooxygenase system and linked its activity to regulation of insulin resistance in liver cells and animal models.

## Results

### BI-1 knock-out disrupts insulin signaling and nutrient metabolism

**Figure 1:**
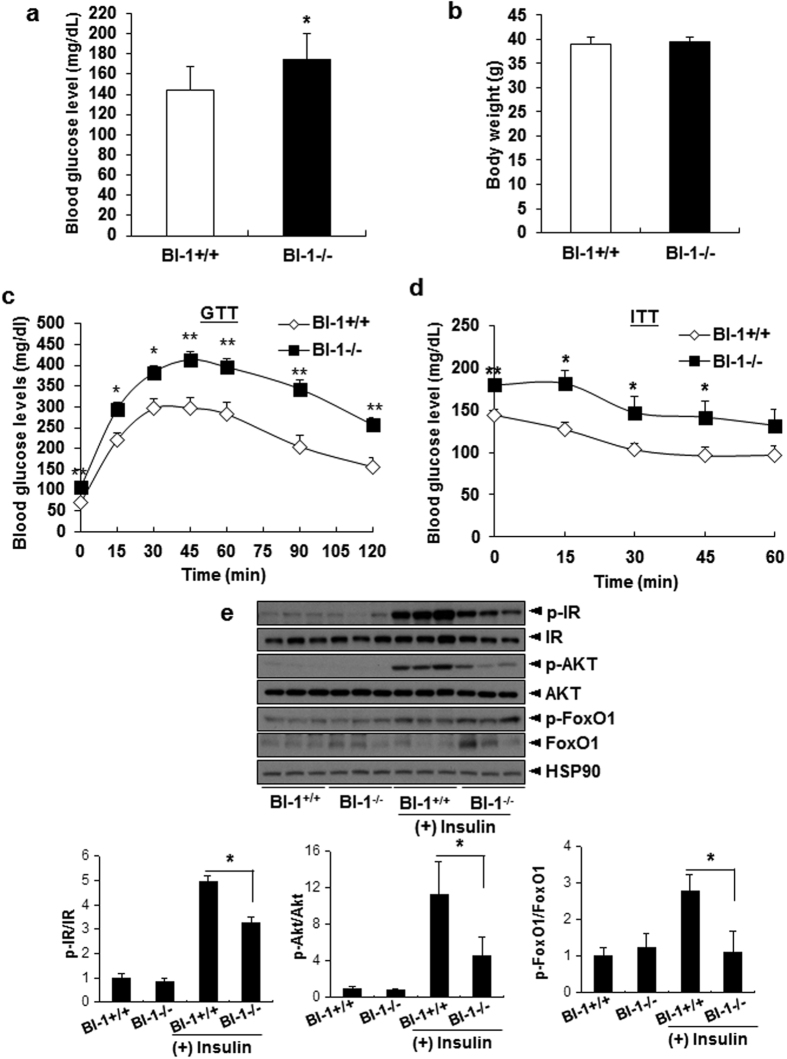
Chronic depletion of BI-1 impairs hepatic glucose metabolism and insulin signaling under high-fat diet conditions. (**a**) Body weight of BI-1 wild-type (BI-1^+/+^) (*n* = 10) and BI-1 knock-out (BI-1^−/−^) male mice (*n* = 11) fed high-fat diet for 8 weeks. (**b**) 6 h fasting glucose levels of high-fat diet-fed mice. (**c**) Glucose tolerance test showing effects of chronic BI-1 depletion on glucose homeostasis. (**d**) Insulin tolerance test showing effects of BI-1 knock-out on insulin sensitivity. (**e**) Western blot analysis showing effects of chronic BI-1 depletion on insulin signaling pathway. Data in (**a**) through (**d**,**e**) represent mean ± SEM (*p < 0.05, **p < 0.005, ***p < 0.0005, *t*-test).

To explore the role of BI-1 in glucose metabolism, glucose and insulin tolerance tests were first performed in normal diet-fed BI-1 wild-type and BI-1 knock-out mice. The results have shown no differences between wild-type and BI-1 knock-out mice, suggesting that BI-1 may not significantly affect glycemia under normal conditions (Fig. S1). To promote diet-induced obesity and insulin resistance, BI-1 wild-type and BI-1 knock-out mice were fed a high-fat diet for 8 weeks. Unlike the normal chow diet-fed conditions, the high fat diet-fed BI-1 knock-out mice exhibited higher plasma glucose levels than wild-type mice (Fig. 1a). No difference in body weight was noted between the two groups (Fig. 1b). Systemic depletion of BI-1 promoted both glucose and insulin intolerance in mice (Fig. 1c,d), and there were no major changes in plasma levels of insulin, triglyceride, and non-essential fatty acid or in hepatic lipid accumulation patterns (Fig. S2a,b). Western blot was performed to verify the integrity of hepatic insulin signaling. Diminished tyrosine phosphorylation of the insulin receptor (IR) and serine 473 phosphorylation of AKT was observed (Fig. 1e). Reduced phospho-FoxO1/FoxO1 ratio was noted in the livers of BI-1 knock-out mice, compared with control mice. Indeed, mRNA levels for genes responsible for gluconeogenesis (Glucose-6-phosphate translocase, Phosphoenolpyruvate carboxykinase, and proliferator-activated receptor-γ coactivator 1α) were higher in the livers of BI-1 knock-out mice than in those of control mice, supporting our hypothesis (Fig. S3).

Despite impaired insulin signaling, elevated mRNA levels of genes involved in fatty acid synthesis (Sterol regulatory element-binding transcription factor 1c, fatty acid synthase, and acetyl-CoA carboxylase) was observed in BI-1 knock-out mice (Fig. S4a), consistent with the mixed insulin resistance phenotype that is often observed in models of type 2 diabetes^20^. Simultaneous induction of genes responsible for triglyceride hydrolysis (hormone-sensitive lipase and adipose triglyceride lipase) and fatty acid beta oxidation (carnitine palmitoyltransferase 1a, medium chain acyl-CoA dehydrogenase, and acyl-CoA oxidase 1) was also observed, suggesting that BI-1 depletion invoked a futile cycle of fat synthesis, hydrolysis, and breakdown in the liver (Fig. S4b,c). There were no significant differences in hepatic expression of genes encoding proinflammatory cytokines between BI-1 wild-type and BI-1 knock-out mice (Fig. S5). No overall differences were observed with respect to food intake and physical activity between BI-1 wild-type and knock-out mice, suggesting that the observed phenotype in BI-1 knock-out mice may not be related to an alteration in overall energy metabolism (Fig. S6).

### BI-1 ameliorates ER stress and its associated CYP2E1 activity and ROS accumulation

**Figure 2:**
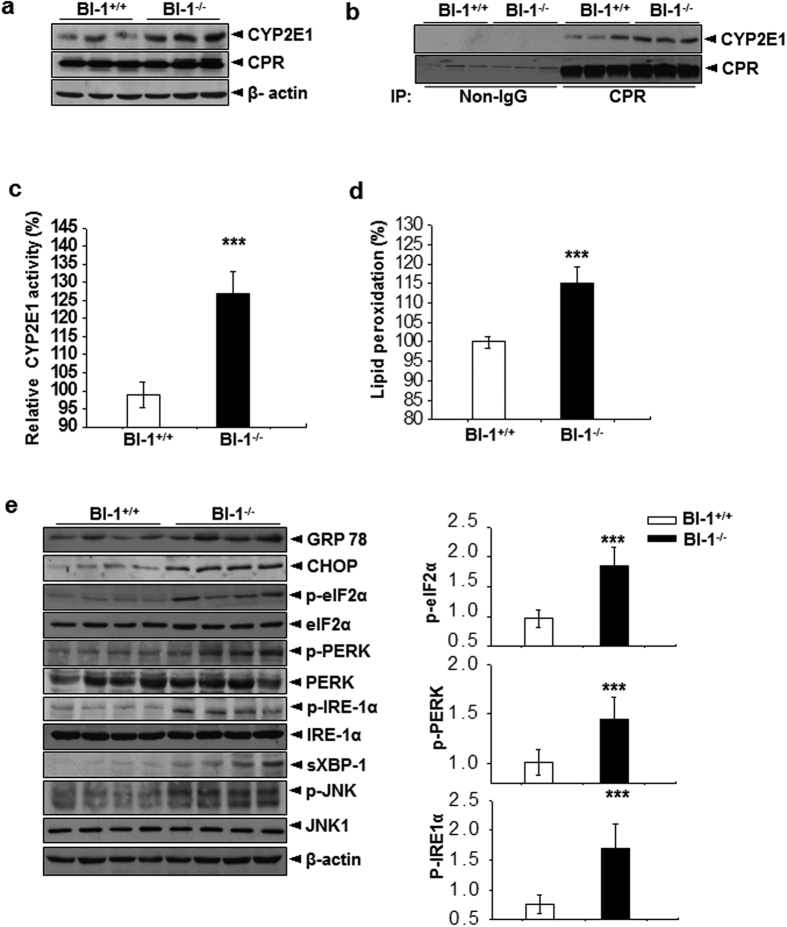
Chronic depletion of BI-1 increases hepatic CYP2E1 activity and promotes hepatic ER stress and ROS accumulation in high-fat diet-fed mice. (**a**) Western blot analysis showing effects of BI-1 depletion on expression levels of CYP2E1 and CPR in livers of BI-1 wild-type (BI-1^+/+^) (*n* = 5) and BI-1 knock-out (BI-1^−/−^) (*n* = 5) mice. (**b**) Co-immunoprecipitation assay of interaction between CPR and CYP2E1 in livers of BI-1 wild-type and BI-1 knock-out mice. (**c**) Estimation of chlorozoxane hydroxylase activity of CYP2E1 and (**d**) lipid peroxidation showing ER-stress induced ROS production in livers of BI-1 wild-type (BI-1^+/+^) (*n* = 5) and BI-1 knock-out (BI-1^−/−^) (*n* = 5) mice. (**e**) Western blot analysis showing effects of chronic depletion of BI-1 on ER stress signaling pathways in livers of high-fat diet-fed mice. Data in (**c**–**e**) represent mean ± SD (*p < 0.05, **p < 0.005, ***p < 0.0005, *t*-test).

CYP2E1 has been suggested to mediate ER stress-associated ROS generation^10^,^21^,^22^. First, increased protein level of CYP2E1 was found in the livers of BI-1 knock-out mice as compared with control mice (Fig. 2a), leading to an increased association with CPR (Fig. 2b). The increased interaction between CYP2E1 and CPR resulted in higher activity of CYP2E1 (Fig. 2c), as well as significant activation of lipid peroxidation in the livers of BI-1 knock-out mice (Fig. 2d), indicating increased ROS generation. Furthermore, the expression of Bip/GRP78, CHOP, phospho-PERK, phospho-eIF-2α and phospho-IRE-1α and its downstream spliced XBP-1 was more enhanced in the livers of BI-1 knock-out mice (Fig. 2e), also showing the recently established role of BI-1, an ER stress regulator.

### Expression of BI-1 regulates insulin signaling and nutrient metabolism through a CYP2E1 and ER stress-associated mechanism

**Figure 3:**
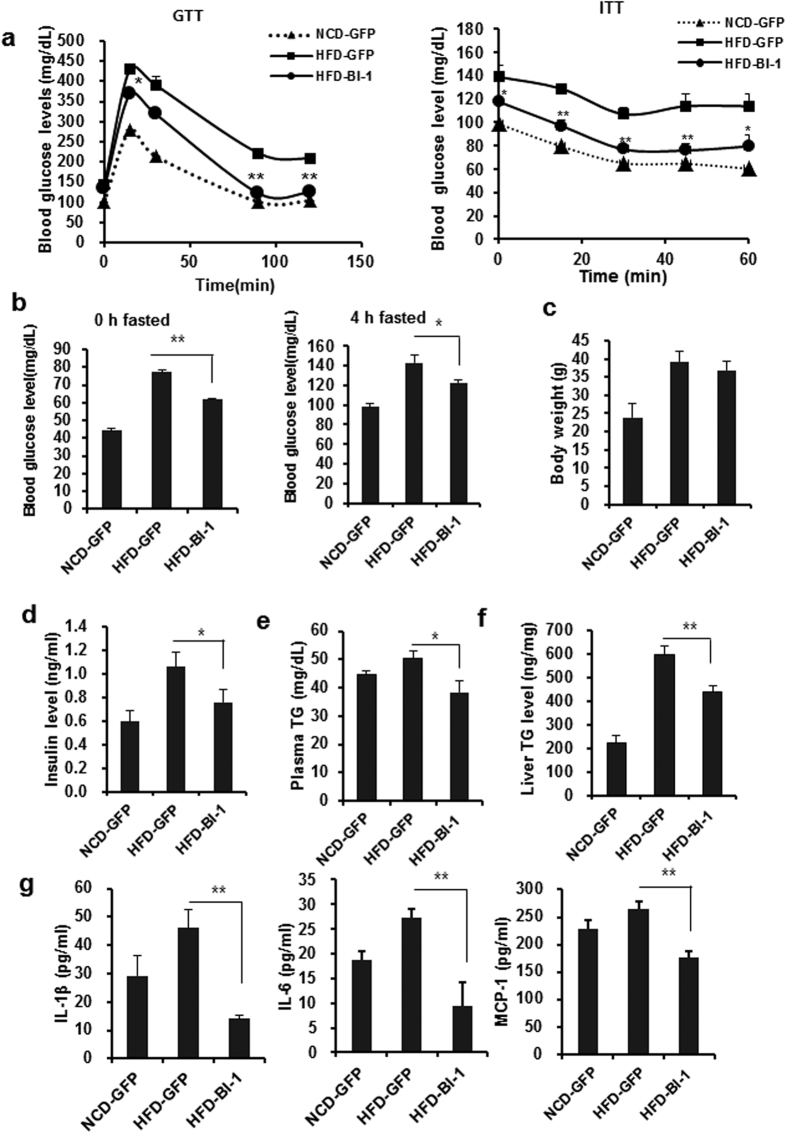
Hepatic BI-1 expression enhances hepatic glucose metabolism and insulin signaling under high-fat diet conditions. (**a**) Mice were fed normal or high-fat diets. Eight weeks later, GFP or BI-1 adenovirus was injected into mice through tail veins. Four days post-injection, glucose and insulin tolerance tests (GTT and ITT) were performed as described in Materials and Methods (*n* = 5–7 each). (**b–g**) Fasting glucose (0 h and 4 h) (**b**), body weight (**c**), plasma insulin (**d**), plasma TG (**e**), liver TG levels, (**f**), and plasma IL-1β, IL-6, and MCP-1 levels (**g**) were measured in normal diet-fed mice infected with GFP virus, high-fat diet-fed mice infected with GFP virus, and high-fat diet-fed mice infected with BI-1 adenovirus (*n* = 6–10 each). Data represent mean ± SEM (*p < 0.05, **p < 0.01, *t*-test).

**Figure 4:**
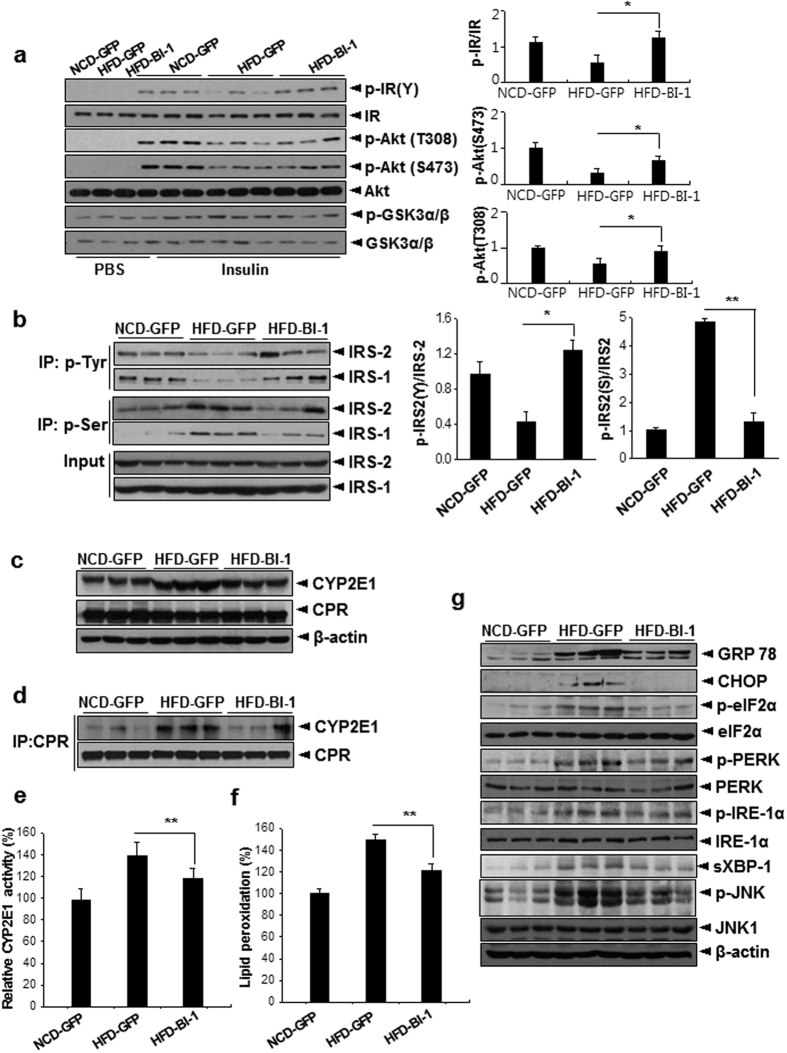
Hepatic BI-1 expression enhances insulin signaling regulation of hepatic CYP2E1 activity, ROS accumulation, and ER stress in high-fat diet-fed mice. (**a**,**b**) Western blot analysis showing effects of BI-1 expression on insulin signaling pathway in normal diet-fed or high-fat diet-fed mice injected with GFP adenovirus alone or with GFP plus BI-1 adenovirus. Quantification of p-IR/IR, p-AKT (S473)/AKT, or p-AKT (T308)/AKT was performed (right). Liver lysates were immunoprecipitated with p-Tyrosine or p-Serine antibody and immunoblotted with antibodies against IRS1/2. Quantification of p-Tyr or Ser IRS2/IRS2 was performed (right). (**c**) Western blot analysis showing effects of BI-1 expression on levels of CYP2E1 and CPR. (**d**) Co-immunoprecipitation assay of interaction between CPR and CYP2E1 in livers of normal diet-fed or high-fat diet-fed mice injected with GFP adenovirus alone or GFP plus BI-1 adenovirus. Estimation of chlorozoxane hydroxylase activity of CYP2E1 (**e**) and lipid peroxidation (**f**) showing ER-stress induced ROS production in livers (each, *n* = 5). (**g**) Western blot analysis showing effects of BI-1 on ER stress signaling pathways in livers of high-fat diet-fed mice (Representative data performed in three separate measurements, *n* = 3–5). Data represent mean ± SD (*p < 0.05, **p < 0.01, *t*-test).

First, adenovirus was injected into normal chow diet-fed mice. Glucose and insulin tolerance did not differ between mice expressing either control GFP or BI-1 (Fig. S7a). On the other hand, hepatic expression of BI-1 ameliorated both glucose and insulin intolerance in high fat diet-fed mice (Fig. 3a). High fat diet-mice expressing BI-1 also displayed reduced fasting blood glucose levels (Fig. 3b) without changes in body weight compared with GFP-expressing high fat diet-fed mice (Figs 3c and S8). Concomitant with changes in blood glucose levels, BI-1 expression in the liver of high fat diet-fed mice reduced plasma insulin levels (Fig. 3d). Both plasma and hepatic TG levels were significantly increased by high fat diet feeding, which was restored to normal levels by hepatic BI-1 expression (Fig. 3e,f). Consistent with this data, H&E staining revealed reduced lipid content in the livers of BI-1-expressing mice compared with controls (Fig. S9). As expected, significant increases in the expression of IL-1β, IL-6, and MCP-1 were observed in those fed a high fat diet compared with controls fed a normal chow diet (Figs 3g and S10). The plasma levels of IL-1β, IL-6, and MCP-1 were all reduced in BI-1-expressing mice compared with controls (Fig. 3g). Furthermore, BI-1 expression also improved insulin signaling in the livers of high fat diet-fed mice, as evidenced by increased tyrosine phosphorylation of IR, IRS-1, and IRS-2, and increased Akt phosphorylation compared with controls (Fig. 4a,b). Serine phosphorylations of IRS-1 and IRS-2 are considered negative feedback markers that inhibit insulin signaling. Significant increases in the serine phosphorylation of both IRS-1 and IRS-2 were observed upon high fat diet-feeding. These levels decreased to nearly normal levels with BI-1 expression.

Protein levels of hepatic CYP2E1 were also reduced in BI-1-overexpressing mice, leading to a decreased association with CPR (Fig. 4c,d). CYP2E1 activity and related lipid peroxidation in the livers of BI-1-overexpressing mice were significantly decreased (Fig. 4e,f). Accordingly, we observed decreased ER stress signaling in BI-1-expressing livers, suggesting the amelioration of ER stress conditions (Fig. 4g).

### BI-1 regulates palmitate-induced insulin resistance through ER stress and associated CYP2E1 activity

**Figure 5:**
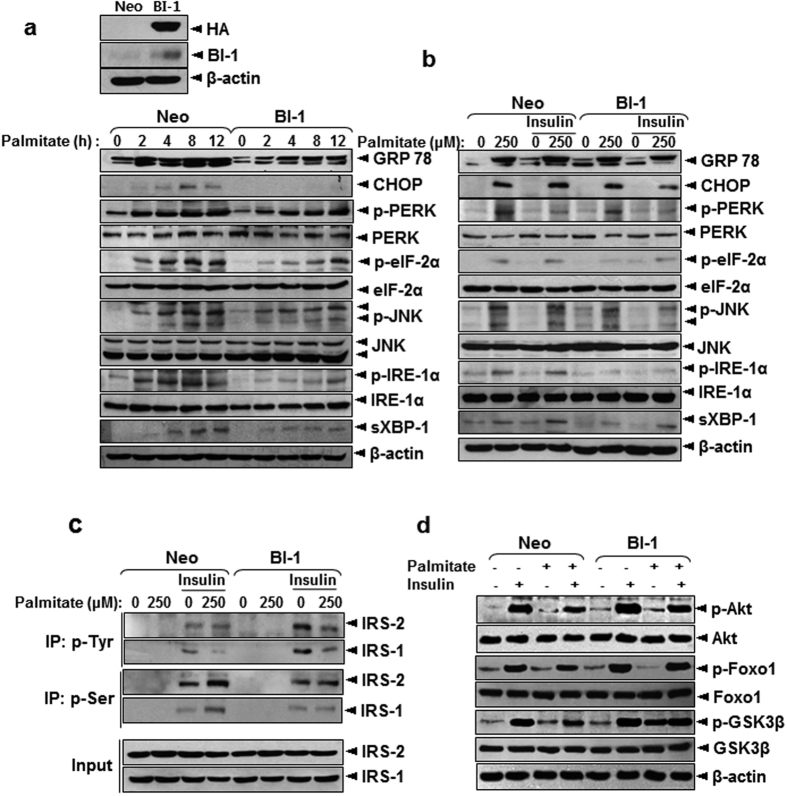
Stable expression of BI-1 in HepG2 cells improves palmitate-induced ER stress and insulin resistance. (**a**) Western blot analysis showing effects of BI-1 expression on palmitate-induced ER stress response. Cells were treated with 250 μM palmitate for indicated periods. Stable expression of BI-1 was confirmed with anti-HA, BI-1, and β-actin (as loading control) antibodies (upper). (**b**) Western blot analysis showing effects of BI-1 expression on palmitate- and insulin-induced ER stress responses. Cells were treated with 250 μM palmitate in presence or absence of insulin. (**c**,**d**) Western blot analysis demonstrating effects of BI-1 expression on palmitate-induced insulin resistance. Cells were treated with 250 μM palmitate in presence or absence of insulin. Cell lysates were immunoprecipitated with p-Tyrosine or p-Serine antibody and immunoblotted with antibodies against IRS1/2 (**c**). Levels of p-Akt, Akt, p-FoxO1, FoxO1, p-GSK3β, and GSK3β are also shown (**d**). (**a**) to (**d**), Representative data performed in three separate measurements.

Having observed the effect of BI-1 depletion on hepatic signaling *in vivo*, we next examined whether expression of BI-1 could induce the similar signaling cascades in cultured hepatocytes. A stable HepG2 hepatic cell line expressing BI-1 (BI-1 cells) and control cells (Neo cells) were generated, and cells were treated with palmitate. Phosphorylation of PERK and activation of its downstream signaling (phospho-eIF-2α) occurred in the presence of palmitate. There was also upregulation of the IRE-1α-dependent pathway upon treatment with palmitate, illustrated by increased expression of sXBP-1 and its downstream target genes (CHOP and GRP78) (Fig. 5a). Increased phospho-JNK was also observed, which could be directly responsible for the observed insulin resistant state induced by palmitate treatment in Neo cells. Unlike Neo cells, BI-1 cells were relatively resistant to palmitate-induced ER stress. Phosphorylation of PERK in response to palmitate treatment in BI-1 cells was less evident than it was in Neo cells and delayed the expression of phospho-eIF-2α compared with Neo cells. The IRE-1α-dependent pathway was comparably less activated by palmitate treatment in BI-1 cells, as shown by the reduced induction of sXBP-1, CHOP, and GRP78 compared with treated Neo cells. Phosphorylation of JNK was also comparably less increased in BI-1 cells than in Neo cells. Taken together, these results suggest that BI-1 modulates the activity of major ER stress pathways (PERK and IRE-1α) in palmitate-treated HepG2 liver cells.

BI-1-associated regulation of ER stress signaling was also examined in response to insulin in palmitate-treated Neo cells and BI-1 cells. As expected, PERK and IRE-1α signaling was activated to a lesser degree in BI-1 cells than in Neo cells (Fig. 5b). To examine whether BI-1 rescues palmitate-induced suppression of insulin signaling, the tyrosine and serine phosphorylation of IRS1/2 was evaluated in response to insulin in palmitate-treated Neo cells and BI-1 cells. Palmitate treatment significantly dampened tyrosine phosphorylation and enhanced serine phosphorylation of IRS1/2 in Neo cells, but not in BI-1 cells, suggesting that expression of BI-1 blocks palmitate-mediated inhibition of insulin signaling (Fig. 5c). BI-1 overexpression also blocked palmitate-induced perturbation of downstream insulin signaling, as indicated by Western blot analysis of phosphorylated Akt, FoxO1, and GSK3 (Fig. 5d). Taken together, these data suggest that BI-1 regulates palmitate-induced insulin resistance primarily through regulation of tyrosine and serine phosphorylation of IRS proteins.

### BI-1 protects against palmitate-induced ER stress and associated CYP2E1 activity

**Figure 6:**
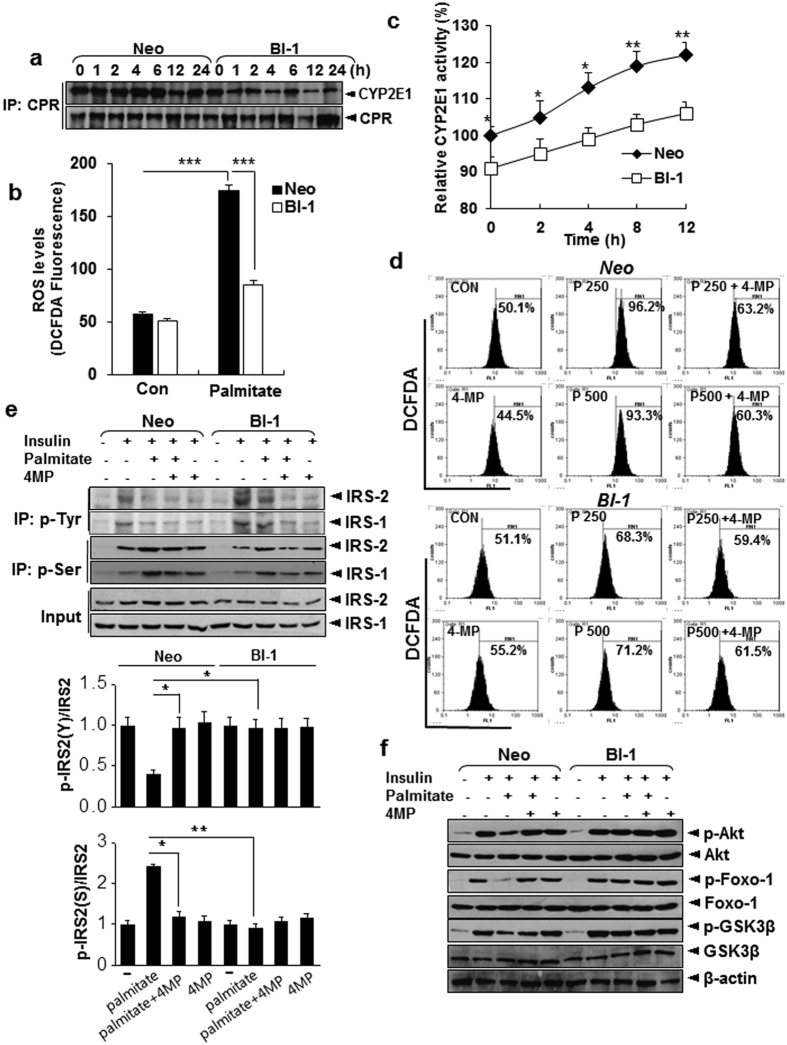
Effects of BI-1 on hepatic ROS production and insulin signaling are mediated by CYP2E1 regulation. (**a**) Co-immunoprecipitation assay demonstrating effects of BI-1 on palmitate-induced interaction of CYP2E1 with CPR. Neo and BI-1 cells were treated with 250 μM palmitate for indicated periods. (**b**) DCF-DA assay revealing palmitate-induced ROS production in Neo and BI-1 cells. Neo and BI-1 cells were treated with 250 μM palmitate for 12 h. DCF-DA, 100 μM, was loaded into cells and fluorescence was measured. (**c**) Chlorozoxane hydroxylation activity assay showing effects of BI-1 expression on CYP2E1 activity. Hepatic microsomes were treated with chlorzoxazone (500 nmol) in a total volume of 1 ml. (**d**) DCF-DA assay showing palmitate-induced ROS production in Neo and BI-1 cells in the presence or absence of 1 mM 4-MP. Palmitate, 250 or 500 μM, was administered to cells for 12 h, then 100 μM DCF-DA was loaded for 30 min. After washing, fluorescence was measured by FACS analysis. (**e**,**f**) Neo and BI-1 cells were treated with or without 1 mM 4-MP, an inhibitor of CYP2E1, in the presence or absence of 250 μM palmitate and/or 100 nM insulin. Western blot analysis showing effects of 4-MP on palmitate-mediated reduction in tyrosine or serine phosphorylation of IRS1 and IRS2 in Neo and BI-1 cells. Cell lysates were immunoprecipitated with p-tyrosine or p-serine 307 antibody and immunoblotted with anti-IRS1 and anti-IRS2 antibodies (**e**). Quantitation of phospho-Tyr and phospho-Ser IRS2. Data represent mean ± SEM. Western blot analysis demonstrating effects of 4-MP on downstream insulin signaling pathway (**f**). Data in (**b**,**c**,**e**) (n = 6) represent mean ± SD (*p < 0.05, **p < 0.005, ***p < 0.0005, *t*-test). (**a**,**d**) and (**f**) Representative data performed in three separate measurements.

**Figure 7:**
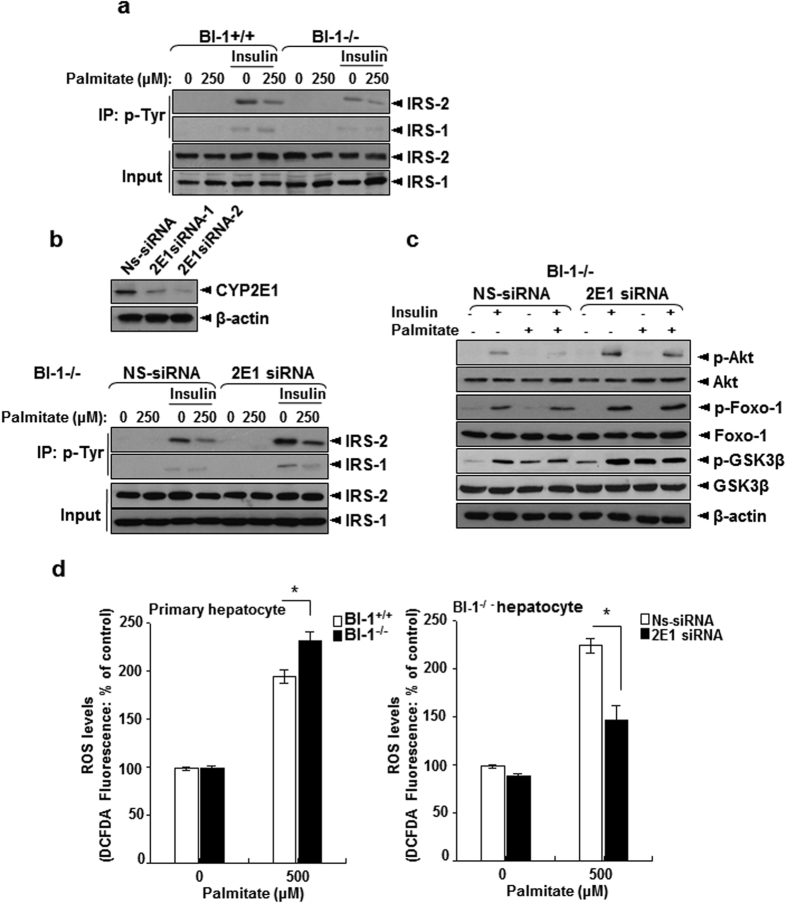
CYP2E1 induces insulin resistance and ROS production in BI-1 knock-out primary hepatocytes. Primary hepatocytes from BI-1 wild-type and BI-1 knock-out mice were treated with 250 μM palmitate in presence or absence of insulin. Cell lysates were immunoprecipitated with p-Tyrosine antibody and immunoblotted with antibodies against IRS1/2 (**a**). Hepatocytes from BI-1 knock-out mice were transiently transfected with non-specific-siRNA or CYP2E1 siRNA. Cells were treated with 250 μM palmitate in presence or absence of insulin. Cell lysates were immunoprecipitated with p-Tyrosine antibody and immunoblotted with antibodies against IRS1/2 (**b**). The Cell lysates were immunoblotted with anti-p-Akt, Akt, p-FoxO1, FoxO1, p-GSK3β, GSK3β or β–actin antibody (**c**). In non-specific-siRNA or CYP2E1siRNA-transfected BI-1 knock-out hepatocytes, 250 μM palmitate was treated for 12 h, then 100 μM DCF-DA was loaded for 30 min. After washing, fluorescence was measured by FACS analysis (**d**). Representative data performed in three separate measurements.

CYP 2E1, a key mediator of ER stress-induced ROS accumulation, was analyzed. Palmitate enhanced the interaction between CPR, CYP2E1, and resulting CYP2E1 activity in Neo cells (Fig. 6a). The induction of ROS by palmitate treatment in BI-1 cells was not as pronounced as in Neo cells (Fig. 6b), indicating that BI-1 also suppresses ROS generation in response to ER stress, perhaps by reducing the activity of CYP2E1 (Fig. 6c). Insulin signaling and ROS generation in response to inhibition of CYP2E1 activity requires confirmation. Treatment with a CYP2E1 inhibitor^23^, 4-methylpyrazole, inhibited the ROS accumulation (Fig. 6d) and restored the palmitate-induced regulation of phospho-IRS2 in the presence of insulin in Neo cells (Fig. 6e), suggesting the essential role of CYP2E1 in palmitate-induced insulin resistance in liver cells. Palmitate-induced reduction of phospho-AKT, phospho-FoxO1, and phospho-GSK3β was also recovered by treatment with 4-methylpyrazole (Fig. 6f). To test the endogenous role of BI-1 at insulin resistance, we applied the same stress, “palmitate at insulin-treated or non-treated condition” in BI-1 knock-out hepatocytes. Expectedly, insulin-dependent increases of tyrosine phosphorylation of both IRS-1 and -2 but there was less prominent in the knock-out cells compared with the control (Fig. 7a). In order to test the impact of CYP2E1 at in the BI-1 knock-out cells, CYP2E1 siRNA was applied to the BI-1 knock-out hepatocytes (Fig. 7b, upper). In the CYP2E1-siRNA-transfected BI-1 knock-out cells, the reduced tyrosine phosphorylation of IRS-1 and -2 was clearly recovered compared with non-specific siRNA-transfected BI-1 knock-out cells. (Figure 7b), and palmitate-induced reduction of phospho-AKT, phospho-FoxO1, and phospho-GSK3β was also recovered at CYP2E1-siRNA-transfected condition (Fig. 7c). ROS analysis was also performed. Consistently, palmitate-induced ROS production was significantly reduced in the CYP2E1 siRNA-transfected BI-1 knock-out hepatocytes compared with non-specific siRNA-transfected BI-1 knock-out cells (Fig. 7d). These data strongly suggest that CYP2E1 is directly responsible for ROS production and the perturbation of hepatic insulin signaling in response to palmitate-induced ER stress. These data also clarify the role of BI-1 in CYP 2E1 expression and activity.

### CYP2E1 enhances palmitate-induced ROS, cell death, and insulin resistance in liver cells

**Figure 8:**
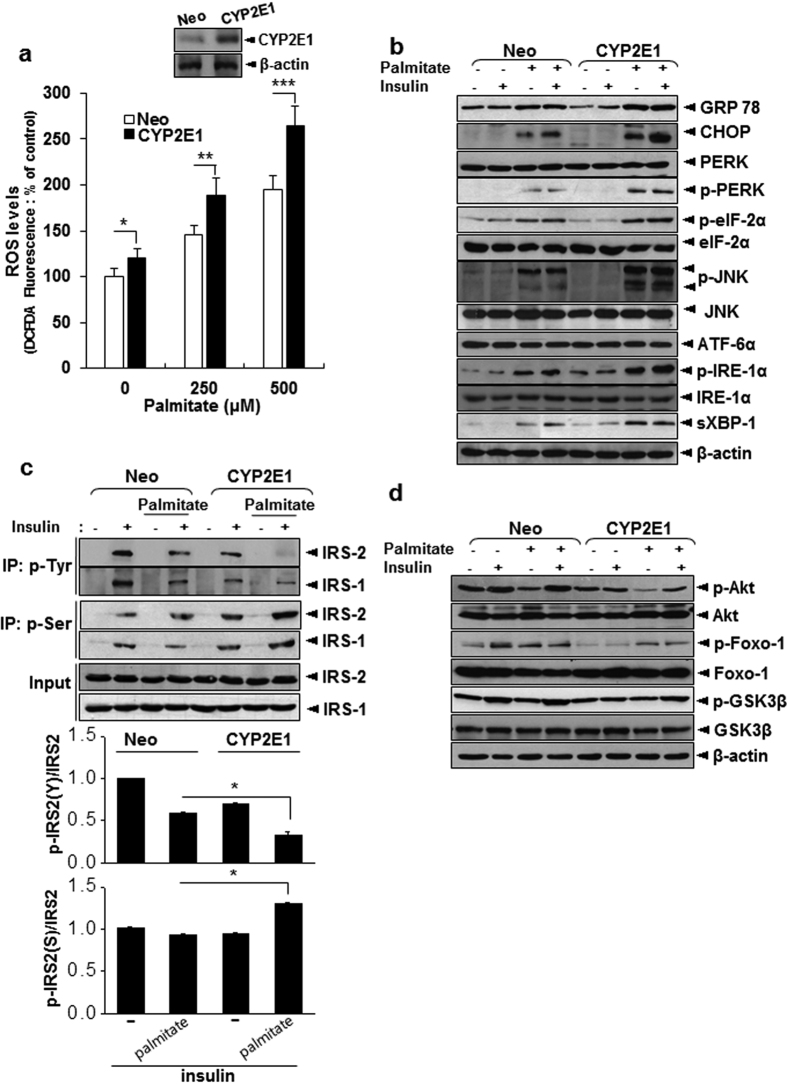
CYP2E1 promotes palmitate-induced ER stress and insulin resistance. (**a**) DCF-DA assay showing palmitate-induced ROS production in Neo and CYP2E1 cells. (**b**) Western blot analysis demonstrating effects of CYP2E1 expression on palmitate-induced ER stress response. (**c**,**d**) Neo and CYP2E1 cells were treated with 250 μM palmitate in presence or absence of 100 nM insulin. Western blot analysis showing effects of CYP2E1 on tyrosine and serine 307 phosphorylation of IRS1 and IRS2, and quantification of tyrosine and serine 307 phosphorylation of IRS2 (**c**) and its downstream proteins (**d**). Data in (**a**,**c**) (n = 4) represent mean ± SD (*p < 0.05, **p < 0.005, ***p < 0.0005, *t*-test). (**b**,**d**), Representative data performed in three separate measurements.

ROS production was higher in HepG2 CYP 2E1 cells than in Neo cells in the basal state, and increases in ROS accumulation in response to palmitate treatment were much more pronounced in CYP2E1 cells than in Neo cells (Fig. 8a). Expression of CYP2E1 enhanced palmitate-induced ER stress and elevated IRE-1-dependent signaling, as shown by enhanced expression of PERK and p-eIF-2α as well as sXBP-1 and p-JNK, respectively (Fig. 8b). Overexpression of CYP2E1 decreased IRS2 phosphorylation both in the presence and absence of palmitate (Fig. 8c). Phosphorylation of AKT, FoxO1, and GSK3β was also decreased in CYP2E1 cells compared with Neo cells (Fig. 8d).

### Bax-1 inhibits the interconnection between CPR and CYP2E1, leading to insulin sensitivity

**Figure 9:**
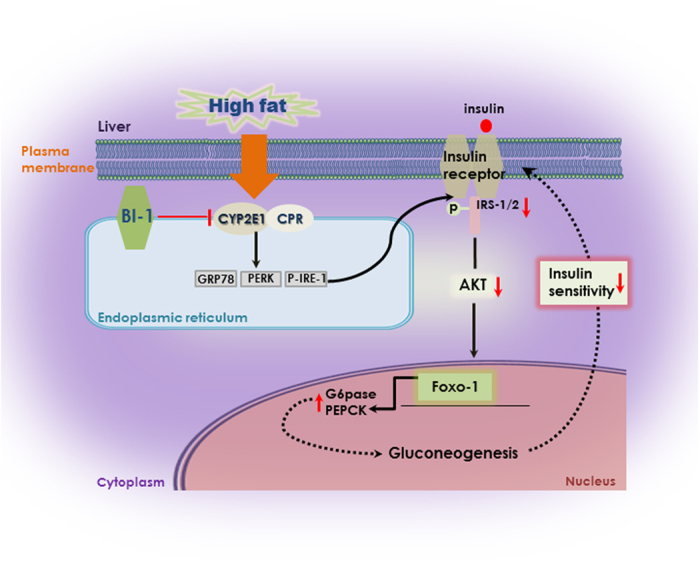
Schematic mechanism of protective role of BI-1 against insulin resistance.

Throughout this study, we focus on ROS involvement in insulin resistance. How BI-1 regulates ROS and insulin resistance is a main theme of this study. The binding between NADPH-dependent monoamine oxidase pathway-lined reductase (CPR) and BI-1, which decreases the interaction between CPR and CYP2E1, is thought to be a mechanism of ROS and related insulin resistance regulation by BI-1. Figure 9 clearly explains the main scheme of BI-1-induced regulation of insulin resistance. However, recent evidence shows that BI-1 is an inhibitor of the IRE1α branch of the UPR, and interferes with IRE1α endonuclease activity^24^. Therefore, we compared the binding status of BI-1 with CPR and IRE-1α. In the absence of treatment, the association of BI-1 with CPR and with IRE-1α was confirmed (Fig. S11). Under free fatty acid stress, the interaction of BI-1 with IRE-1α was less stable compared with that of BI-1 with CPR. Although binding intensity might be not a critical parameter explaining biological function, the stable association of BI-1 with CPR is suggested to be a regulatory mechanism of BI-1 against ROS and insulin resistance.

## Discussion

Our previous studies suggested that BI-1 is an ER stress regulator that also inhibits the activity of CYP2E1. This study identified CYP2E1 as a crucial player in ER stress-induced ROS production, supporting our hypothesis that the antagonistic activity of BI-1 against ER stress-induced insulin resistance in liver cells is due to inhibition of ROS via interconnection between CPR and CYP2E1. The BI-1 knock-out mouse results also showed that BI-1 can function to inhibit ER stress-induced insulin resistance.

Electron uncoupling between CYP2E1 and CPR has been considered a source of ROS^8^ potentially linked to the dysregulation of insulin resistance in hepatocytes^25^. In addition, increased infusion of saturated fat into the liver may be critical in promoting insulin resistance in this organ^26^,^27^. Insulin resistance leads to a spectrum of non-alcoholic fatty liver diseases, with manifestations ranging from pure steatosis to non-alcoholic steatohepatitis, which can evolve to cirrhosis^27^,^28^. In the presence of infused saturated fat, liver cells are continuously subject to insults from ROS (reactive oxygen species). These include free radicals such as the superoxide radical (O_2_^−^) and H_2_O_2_, a common feature of non-alcoholic fatty liver diseases.

BI-1, an ER stress regulator, increased insulin sensitivity via regulation of IRS-2 (p-Tyr and p-Ser) phosphorylation in free fatty acid-exposed liver cells (Fig. 5c). Non-alcoholic fatty liver diseases patients have increased lipolysis and increased delivery of free fatty acids to the liver. Higher free fatty acid concentrations are associated with more severe liver disease^29^,^30^. In *in vitro* approaches, palmitate has been used to study the effect of free fatty acids on hepatic signaling by mimicking conditions of hepatic diseases such as non-alcoholic fatty liver diseases. ER stress and JNK activation have been suggested as major instigators of insulin resistance, a main component of the non-alcoholic fatty liver diseases-associated phenomena^31^,^32^,^33^,^34^. Palmitate and high-fat diet conditions were found to stimulate IRE-1α and PERK signaling transduction (Figs 2e,4g and 5a), among other ER stress proteins. Similarly, the two arms of ER stress signaling mentioned above have been suggested to be main mechanisms of lipid metabolism disturbance and insulin sensitivity dysregulation^35^,^36^,^37^. In contrast to the results of multiple ER stress-involvement studies, in mice placed on a 3-day high fat diet, one specific ER stress arm, eIF2α signaling, exhibited high levels of induced hepatic lipid accumulation and insulin resistance^30^. Recently, apoB100 protein synthesis was studied in relation to one branch of the ER stress pathways, PERK activation^38^. Bailly-Maitre B *et al*. demonstrated that HFD induces only a single arm of ER stress, IRE-1α, and its related insulin resistance^29^, and inhibition of this arm by BI-1 expression has been suggested previously, in contrast to the results of this study. The interaction of BI-1 with IRE-1α^24^,^39^ is considered a mechanism against insulin resistance. Our study also included the association between IRE-1α and BI-1 (Fig. S11). Considering that the interaction between BI-1 and IRE-1α was diminished after relatively long time periods (more than 6 hours of treatment), the interaction of IRE-1α with BI-1 may not be stable under free fatty acid stress, in contrast to the stable interaction between CPR and BI-1. The regulation effect of BI-1 on insulin resistance signaling was clearly shown at the 12-hour time point (Figs 5c and 6e), when a slight binding affinity with IRE1α was observed. Possible competition between CPR and IRE-1α for association with BI-1 should be considered. This study suggests that the stable binding of BI-1 with CPR is another regulatory mechanism of insulin resistance, in addition to IRE1-α. BI-1 binds to CPR to dissociate it from CYP2E1 and subsequently inhibit ROS production, suggesting that BI-1-mediated inhibition of CYP2E1 activity may decrease ROS production and ER stress. CYP2E1 knock-out mice displayed consistent, substantial protection against high fat diet-induced hepatic insulin resistance and insulin-stimulated adipose tissue glucose uptake, providing evidence that CYP2E1 is a negative regulator of glucose and energy metabolism^40^,^41^,^42^. Increased oxidative stress has been implicated as a causative factor in insulin resistance, and considerable increases in oxidative stress markers in diabetes and related conditions have also been reported^43^. Mitochondria-associated ROS are involved in the dysregulation of lipid metabolism and insulin resistance^44^,^45^, although no impairments in mitochondrial function have been observed with the activation of the two major ER stress pathways (PERK/eIF2α and IRE1/XBP1) in a non-alcoholic fatty liver disease model^46^. Recently, the amplified ROS connection between mitochondria and ER has also attracted attention in this field^47^,^48^, indicating that the subcellular organelle-connected transfer/communication of ROS signaling might be interrelated, ultimately leading to biological changes including cellular dysmetabolism and insulin resistance^49^,^50^. Interestingly, a significant amount of CYP2E1 has been suggested to be localized within liver mitochondria, inducing mitochondrial dysfunction and insulin resistance and also suggesting the possibility of connected ROS signaling between the ER and mitochondria through CYP2E1^12^. Through CYP2E1, intra-ER oxidative stress has been also reported, suggesting that ER protein-folding machinery capacitance might be affected, leading to ER stress and related physio/pathological changes, particularly hepatic lipid dysmetabolism^51^.

CYP2E1 induction of oxidative stress impairs insulin signaling to IRS-1, IRS-2, PI3-kinase, and Akt/PKB^42^,^52^‒^55^ and the CYP2E1-mediated oxidative stress is one of the key mechanisms for ER dysfunction^56^. The CYP2E1 is directly or indirectly linked to intra-ER altered protein folding, where ROS accumulation is suggested to alter ER folding^57^,^58^. CPR, ER-localized NADPH-dependent oxygenase (NOX4)^59^ and other NADPH-linked oxidative folding machinery such as protein disulfide isomerase (PDI) and endoplasmic reticulum oxidoreductase (ERO)^60^ are possible candidate proteins for intra-ER-originating ROS-linked protein folding alterations, which lead to ER stress and hepatic dyslipidemic insulin resistance. In our model, a high fat diet might induce conditions similar to xenobiotic treatment conditions in which CYP machinery is involved. The main factor for the mentioned proteins-associated ER-redox coupling/uncoupling seems to be NADPH. With intra-ER coupling of NADPH, the PDI/ERO1α-oxidoreduction process also amplifies CYP initiation of ROS production, which induces protein aggregation and the ER stress response^8^. Another basic characteristic of BI-1, the Ca^2+^ leaky channel effect, may be considered a potential mechanism involved in palmitate-induced ROS and insulin resistance. ER calcium release and resultant mitochondrial dysfunction have been suggested as a mechanism involved in palmitate-induced ROS^22^. It has been also suggested that insulin resistance depends on intracellular Ca^2+^ and ROS^10^,^61^.

In summary, we showed that BI-1 overexpression protects hepatocytes from ROS accumulation and insulin resistance in response to palmitate-induced ER stress conditions. Knock-out of BI-1 in mice exacerbates glucose intolerance and impairs insulin signaling induced by high-fat diet-induced chronic ER stress. The regulatory role of BI-1 is mediated by inhibition of CYP2E1 expression and interaction between CPR and CYP2E1. These data suggest that BI-1 may serve as a novel therapeutic target protein for the treatment of ER stress-induced hepatic insulin resistance.

## Materials and Methods

### Materials

Palmitate was purchased from Calbiochem (San Diego, CA, USA). DCF-DA (2′,7′-dichlorofluorescin diacetate) was obtained from Molecular Probes (Eugene, OR, USA). Antibodies against β-actin, GRP78, CHOP, ATF6α, p-PERK, sXBP-1, p-JNK, Akt, Foxo1, GSK3β, IRS-1, IRS-2, HSP90, and CPR were acquired from Santa Cruz Biotechnology (Santa Cruz, CA, USA). Antibodies against p-AKT, p-Foxo-1, Foxo-1, p-AKT, p-GSK3β, p-Tyr, p-IR, and IR were acquired from Cell Signaling Technology (Beverly, MA, USA). Chlorozoxazone and p-nitrophenol were purchased from Sigma-Aldrich Co. (St. Louis, MO, USA).

### Animal experiments

BI-1 wild type (BI-1^+/+^) and BI-1 knock-out (BI-1^−/−^) mice were obtained from Dr. John C. Reed at the Stanford-Burnham Institute for Medical Research (La Jolla, CA, USA). All animal experiments were carried out in accordance with the National Research Council’s Guidelines (IACUC, Knock-outrea) for the Care and Use of Laboratory Animals. The experimental protocol was approved by the Animal Experiments Committee of Chonbuk National University Laboratory Animal Center (CBU 2013-0015).

Animals were housed in standard 75-in2 static cages. The cages and bedding were autoclaved prior to use. All husbandry practices aside from housing density were in accordance with the Knock-outrean Association for Laboratory Animal Science Guide. Cages were changed once or twice per week, depending on need. To induce obesity and insulin resistance, 8-week-old male C57BL/6, BI-1 wide-type and BI-1 knock-out mice were fed a high-fat diet (60 kcal% fat diet: D12492 of Research Diets) for 8 weeks. Animal experiments involving adenoviruses were performed as described previously^62^. To measure fasting blood glucose level, animals were fasted for 16 h or 6 h with free access to water. For glucose tolerance test (GTT), 16 h-fasted mice were injected intraperitoneally with glucose (1.5 g/kg of body weight for high-fat diet). For insulin tolerance test (ITT), 6 h-fasted mice were injected intraperitoneally with 0.5 unit/kg (high-fat diet) body weight of insulin. Blood glucose levels were measured from tail vein blood collected at the designated time.

### Recombinant adenoviruses

Adenoviruses expressing GFP only and BI-1 were generated by homologous recombination between adenovirus backbone vector pAD-Easy and linearized transfer vector pADTrack. For animal experiments, viruses were purified on a CsCl gradient, dialyzed against PBS buffer containing 10% glycerol, and stored at −80 °C.

### Histology

Histology works were performed on frozen liver sections from BI-1 wide-type (*n* = 6) and BI-1 knock-out (*n* = 6) mice. Hematoxylin and eosin (H&E) stains showed nuclei and cytoplasm in liver tissue. Oil-red O staining on these frozen liver sections were performed for detecting lipid droplets.

### Quantitative PCR

Total RNA from liver tissue was extracted using Easy-spin total RNA extract kit (Qiagen). 1 mg of total RNA was used for generating cDNA with amfiRivert reverse transcriptase (GenDEPOT), and was analyzed by quantitative PCR using SYBR green PCR kit and Bio-Rad Real Time System. All data were normalized to expression of ribosomal L32.

### Measurement of metabolites

Blood glucose levels were determined from tail vein blood using an automatic glucose monitor (One Touch; LifeScan, Inc.). Plasma triglyceride and non-essential fatty acid were measured accordingly with the protocols of the colorimetric assay kits (Waknock-out). Plasma insulin was measured accordingly with the protocols of the Mouse Insulin ELISA Kit (ALPCO). Total liver lipids were extracted with chloroform-methanol (2:1, v/v) mixture as described previously^26^.

### Generation of Neo and BI-1 stable cell lines

HA-pcDNA3-BI-1 and Neo-pcDNA3 plasmids (Neo: containing neomycin-selection marker) were stably transfected into 80% confluent HepG2 cells with lipofectamine (Invitrogen, CA). G418 (1 mg/mL; Waknock-out) was used for selection. Neo and BI-1 stably transfected HepG2 cell lines were generated and used during this study.

### DCF-DA assay

Intracellular ROS levels were measured as previously described^27^. After treatment of palmitate with or without 4-MP or palmitate with or without insulin, the cells were incubated with 100 μM 2′,7′-dichlorofluorescin diacetate (DCF-DA) at 37 °C for 30 min. The fluorescence intensity of 2′,7′-dichlorofluorescein formed by a reaction between DCF-DA and intracellular ROS was analyzed by PAS flow cytometry (Partec, Münster, Germany) at excitation and emission wavelengths of 488 and 525 nm, respectively. Data are expressed as representative histograms from three independent experiments.

### Microsomal fractionation

The microsomal fraction was obtained as previously described^8^. Briefly, the cells were re-suspended in buffer A (250 mM sucrose, 20 mM HEPES, pH 7.5, 10 mM KCl, 1.5 mM MgCl_2_, 1 mM EDTA, 1 mM EGTA, and 1× protease inhibitor complex (Roche Diagnostics, Mannheim, Germany) on ice for 30 min. The cells were homogenized and the lysates centrifuged at 750 *g* for 10 min at 4 °C to remove the non-lysed cells and nuclei. The supernatant was then centrifuged at 100,000 *g* for 1 h at 4 °C. The resulting supernatant was discarded, and the pellet was saved as the light membrane (LM:ER/microsome) fraction.

### Western blotting

For Western blotting, whole-cell lysates were generated by using a buffer consisting of 1% Nonidet P-40, 50 mM HEPES (pH 7.5), 100 mM NaCl, 2 mM EDTA, 1 mM pyrophosphate, 10 mM sodium orthovanadate, 1 mM phenylmethylsulfonyl fluoride, and 100 mM sodium fluoride. Equal amounts of lysates were subjected to sodium dodecyl sulfate-10% polyacrylamide gel electrophoresis and then transferred to Immobilon-P membranes (Millipore) in transfer buffer (25 mM Tris, 192 mM glycine, 20% [vol/vol] methanol). Membranes were first rinsed in Tris-buffered saline (TBS: 10 mM Tris [pH 7.4], 150 mM NaCl) and then blocked overnight at room temperature in TBS-5% bovine serum albumin (BSA). Various antibodies were used at a dilution of 1:1000 in TBS-5% BSA. Antibody-antigen complexes were detected with horseradish peroxidase-conjugated protein A or horseradish peroxidase-conjugated goat anti-rabbit immunoglobulin G (Bio-Rad) and a chemiluminescent substrate development kit (Amersham, CA, USA). Equal loading was ascertained by the presence of β-actin.

### Immunoprecipitation

Immunoprecipitation was performed as previously described^1^. For immunoprecipitation, cell lysates were prepared in 50 mM Tris-HCl, pH 8.0, containing 150 mM NaCl, 0.015% phenylmethylsulfonyl fluoride, 1 mM dithiothreitol, 1 mM EDTA, 1% sodium deoxycholate, 1% Triton, and 1% SDS. Cell lysates were incubated at 70 °C for 15 min to ensure complete cell lysis and diluted with lysis buffer to achieve SDS concentration of 0.1% (w/v), as reported. Media were collected and centrifuged at 13,000 rpm for 10 min to remove cell debris. Supernatants (0.9 ml) were combined with 0.1 ml of 10 X lysis buffer containing 1% SDS. Cell lysates and media were incubated with antibodies (1:100 dilution) for 2 h and then with 20 μl of protein A/G-Sepharose (10% solution) for an additional 2 h. Immuno-complexes were collected by centrifugation, washed three times with 10 mM Tris, pH 7.5, containing 0.1 M NaCl and 1% Triton, eluted in 50 μl of sample buffer, separated on SDS-polyacrylamide gel, and Western blotting were performed as described in the section of Western blotting.

### CYP 2E1 siRNA transfection

Primary mouse hepatocytes were isolated from BI-1 wide-type and BI-1 knock-out by collagenase digestion, following a previously described protocol^63^. Scramble, CYP2E1-1, and CYP 2E1-2 siRNAs were purchased from Bioneer (Bioneer, Daejeon, Korea). The scramble-sense siRNA targeted the sequence 5′-CCUACGCCACCAAUUUCGU-3′, and the scramble-antisense siRNA targeted the sequence 5′-CGAAAUUGGUGGCGUAGG-3′. CYP 2E1-1-sense siRNA targeted the sequence 5′-GAUUACGAUGACAAGAAGU (dTdT)-3′, and CYP 2E1-1-antisense siRNA targeted the sequence 5′-ACUUCUUGUCAUCGUAAUC (dTdT)-3′. CYP 2E1-2-siRNA targeted the sequence 5′-UCAACCUCGUCCCUUCCAA (dTdT)-3′, and CYP 2E1-2-siRNA -antisense siRNA targeted the sequence 5′-UUGGAAGGGACGAGGUUGA (dTdT)-3′. For transient transfection, cells were plated to obtain 70–80% confluence in 10-cm dish. After overnight incubation, plated primary hepatocytes were transfected with 50 nM of scramble (negative control) siRNA, CYP 2E1-1, or CYP 2E1-2 using Lipofectamine® 3000 (Invitrogen, Carlsbad, CA, USA). After 24 h of transfection, fresh medium was exchanged.

### Statistical Analysis

Statistical differences were evaluated by analysis of variance (ANOVA) in acidity level-response experiments and two-tailed Student’s *t*-tests. In each case, the statistical test used is indicated, and the number of experiments is stated individually in the legend of each figure.

## Additional Information

**How to cite this article**: Lee, G.-H. *et al*. Effect of BI-1 on insulin resistance through regulation of CYP2E1. *Sci. Rep.* **6**, 32229; doi: 10.1038/srep32229 (2016).

## Supplementary Material

Supplementary Information
