# Author Correction: Effect of BI-1 on insulin resistance through regulation of CYP2E1

**DOI:** 10.1038/s41598-018-37594-4

**Published:** 2018-12-20

**Authors:** Geum-Hwa Lee, Kyoung-Jin Oh, Hyung-Ryong Kim, Hye-Sook Han, Hwa-Young Lee, Keun-Gyu Park, Ki-Hoan Nam, Seung-Hoi Koo, Han-Jung Chae

**Affiliations:** 1 Department of Pharmacology and New Drug Development Institute, Medical School, Chonbuk National University, Jeonju, 561-181 Republic of Korea; 2 Division of Life Sciences, Korea University, 145 Anam-Ro, Seongbuk-Gu, Seoul, 136-713 Republic of Korea; 3 Metabolic Regulation Research Center, Korea Research Institute of Bioscience and Biotechnology (KRIBB), Daejeon, 305-806 Republic of Korea; 4 Department of Dental Pharmacology and Wonkwang Dental Research Institute, School of Dentistry, Wonkwang University, Iksan, 570-749 Republic of Korea; 5 Department of Internal Medicine, Kyungpook National University School of Medicine, Daegu, 700-721 Republic of Korea; 6 Laboratory Animal Resource Center, KRIBB, Ochang-eup, 363-883 Republic of Korea

Correction to: *Scientific Reports* 10.1038/srep32229, published online 31 August 2016

This Article contains an error in the labelling of the middle and lower panels of Supplementary Figure 11, which were incorrectly labelled as ‘IP: IRE-1α’ and ‘IP: CPR’ respectively. Both panels should be labelled as ‘IP:HA’. In addition, the ‘IRE-1α’ and ‘HA’ labels in the middle panel and the ‘CPR’ and ‘HA’ labels in the lower panel were inverted. The correct Supplementary Figure 11 appears below as Figure 1.

**Figure 1:**
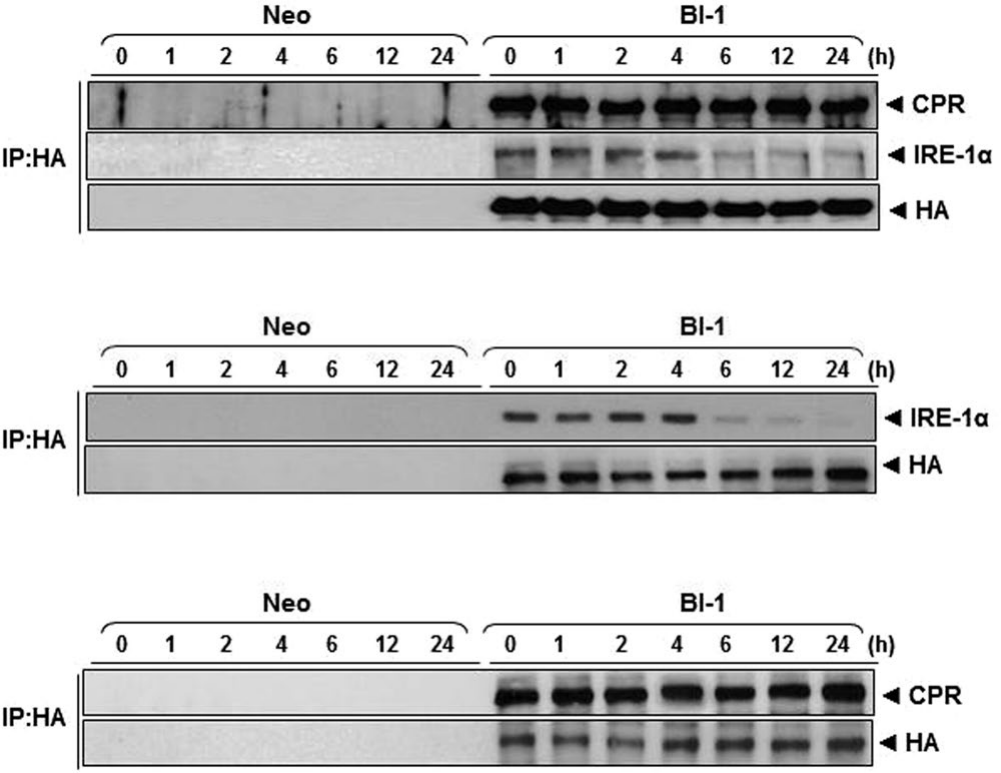
The interaction of BI-1 with CPR and IRE-1α. Immunoprecipitation assay demonstrating the effects of BI-1 on palmitate-induced interactions with CPR or IRE-1α. Neo and BI-1 cells were treated with 250 µM palmitate for the indicated periods. Immunoprecipitation was performed with anti-HA, IRE-1α, or CPR antibody. Western blotting was performed with anti-CPR, IRE-1α or HA antibody. CPR, NADPH-dependent CYP reductase.

