# Supplementary material for: Effect of BI-1 on insulin resistance through regulation of CYP2E1
Source: Sci Rep. 2016 Aug 31;6:32229. doi: 10.1038/srep32229 (PMC5006057; doi:10.1038/srep32229)
Supplement: Supplementary Information [file srep32229-s1.pdf]

Effect of BI-1 on insulin resistance through regulation of CYP2E1

Geum-Hwa Lee[1¶], Kyoung-Jin Oh[2, 3¶], Hyung-Ryong Kim[4¶], Hye-Sook Han[2], Hwa-Young Lee[1], Keun-Gyu Park[5], Ki-Hoan Nam[6], Seung-Hoi Koo[2]*, Han-Jung Chae[1]*

[1]Department of Pharmacology and New Drug Development Institute, Medical School, Chonbuk National University, Jeonju, 561-181, Republic of Korea; [2]Division of Life Sciences, Korea University, 145, Anam-Ro, Seongbuk-Gu, Seoul, 136-713, Republic of Korea; [3]Functional Genomics Research Center, Korea Research Institute of Bioscience and Biotechnology (KRIBB), Daejeon, 305-806, Republic of Korea, [4]Department of Dental Pharmacology and Wonkwang Dental Research Institute, School of Dentistry, Wonkwang University, Iksan, 570-749, Republic of Korea, [5]Department of Internal Medicine, Kyungpook National University School of Medicine, Daegu, 700-721, Republic of Korea, [6]Laboratory Animal Resource Center, KRIBB, Ochang-eup, 363-883, Republic of Korea

*Corresponding author

Seung-Hoi Koo (koohoi@korea.ac.kr); Division of Life Sciences, Korea University, 145 Anam-Ro, Seongbuk-Gu, Seoul, 136-713, Korea, Tel: +8-2-3290-3403, Fax: +82-2-3290-4144

Han-Jung Chae (hjchae@jbnu.ac.kr); Department of Pharmacology, School of Medicine, Chonbuk National University; Jeonju, 560-182; Republic of Korea; Tel: + 82-63-270-3092; Fax: 82-63-275-2855

[¶]These authors contributed equally to this work.

Supplementary Figure 1. Effects of BI-1 knockout on glucose and insulin tolerance test results. BI-$1^{+/+}$ (*n*=6) and BI-$1^{-/-}$ male mice (*n*=9) were fed a normal diet. Glucose and insulin tolerance tests (GTT and ITT) were performed as described in Materials and Methods.

Supplementary Figure 2. Effect of BI-1 knockout on plasma lipid profiles in mice. (a) Changes in plasma insulin (left), TG (middle), and NEFA levels (right) of BI-$1^{+/+}$ (*n*=10) and BI-$1^{-/-}$ male mice (*n*=11) fed a high-fat diet. (b) H&E staining and Oil Red O staining to determine the effects of BI-1 knockout on hepatic TG accumulation. Data in (a) and (b) represent mean ± SEM.

Supplementary Figure 3. Effect of BI-1 knockout on glucogenic genes in mice. qPCR analysis of expression levels of BI-1 and gluconeogenic genes in livers of BI-$1^{+/+}$ (*n*=5) and BI-$1^{-/-}$ male mice (*n*=5). Data represent mean ± SEM (*: p<0.05, **: p<0.005, ***: p<0.0005, *t*-test).

Supplementary Figure 4. Effects of BI-1 knockout on lipogenic gene profiles in mice. (a) qPCR analysis showing expression levels of lipogenic genes in livers of BI-$1^{+/+}$ (*n*=5) and BI-$1^{-/-}$ male mice (*n*=5). (b) qPCR analysis of expression levels of lipolytic genes in livers of BI-$1^{+/+}$ (*n*=5) and BI-$1^{-/-}$ male mice (*n*=5). (c) qPCR analysis of expression levels of fatty acid oxidation genes in livers of BI-$1^{+/+}$ (*n*=5) and BI-$1^{-/-}$ male mice (*n*=5). Data in (a) - (c) represent mean ± SEM (*: p<0.05, **: p<0.005, ***: p<0.0005, *t*-test).

Supplementary Figure 5. Effect of BI-1 knockout on proinflammatory gene expression in mice. qPCR analysis showing expression levels of proinflammatory genes in livers of BI-$1^{+/+}$ (*n*=5) and BI-$1^{-/-}$ male mice (*n*=5).

Supplementary Figure 6. Effect of BI-1 knockout on food intake and physical activity in mice. (a)-(e) Food intake (a), food consumption rate (b), drink consumption (c), drink consumption rate (d), and locomotor activity (e) were measured in BI-$1^{+/+}$ and BI-$1^{-/-}$ mice during high-fat diet consumption using metabolic cages (*n*=6 each). Data in (a)-(e) represent mean ± SEM.

Supplementary Figure 7. Effect of BI-1 expression on glucose and insulin tolerance tests. Mice were fed normal calorie diets. GFP or BI-1 adenovirus was injected into mice through tail veins. Four days later, glucose and insulin tolerance tests (GTT and ITT) were performed as described in Materials and Methods. GFP, normal calorie diet-fed GFP virus-infected mice, BI-1, normal calorie diet-fed BI-1 virus-infected mice.

Supplementary Figure 8. Effect of BI-1 expression on body weight change. Mice were fed a normal diet or high-fat diet for 8 weeks. GFP or BI-1 adenovirus was injected into mice through the tail vein. Body weight was measured from mice either before adenoviral injection or 4-days post-adenoviral injection. Data represent mean ± SD (*t*-test). NCD-GFP, normal calorie diet-fed GFP vector-infected mice, NCD-BI-1, normal calorie diet-fed BI-1 with GFP vector-infected mice, HFD-BI-1, high fat diet-fed BI-1 virus-infected mice.

Supplementary Figure 9. Effect of BI-1 expression on hepatic lipid accumulation. Mice were fed a normal diet or high-fat diet for 8 weeks. GFP or BI-1 adenovirus was injected into mice through tail veins. Five days later, mice were sacrificed and livers were stained with H&E. Images were captured by light microscopy at 400X. NCD-GFP, normal calorie diet-fed GFP vector-infected mice, NCD-BI-1, normal calorie diet-fed BI-1 with GFP vector-infected mice, HFD-BI-1, high fat diet-fed BI-1 virus-infected mice.

Supplementary Figure 10. Effects of BI-1 expression on pro-inflammatory cytokine mRNA levels. qPCR analysis showing expression levels of proinflammatory genes in livers of normal diet-fed GFP virus-injected (n=5), high-fat diet-fed GFP virus-injected mice (n=5), or high-fat diet-fed BI-1 virus-injected mice (n=5). Data represent mean ± SD (*: $p<0.05$, **: $p<0.01$, *t*-test). NCD-GFP, normal calorie diet-fed GFP vector-infected mice, NCD-BI-1, normal calorie diet-fed BI-1 with GFP vector-infected mice, HFD-BI-1, high fat diet-fed BI-1 virus-infected mice.

Supplementary Figure 11. The interaction of BI-1 with CPR and IRE-1α. Immunoprecipitation assay demonstrating the effects of BI-1 on palmitate-induced interactions with CPR or IRE-1α. Neo and BI-1 cells were treated with 250 μM palmitate for the indicated periods. Immunoprecipitation was performed with anti-HA, IRE-1α, or CPR antibody. Western blotting was performed with anti-CPR, IRE-1α or HA antibody. CPR, NADPH-dependent CYP reductase

Sup Fig 1

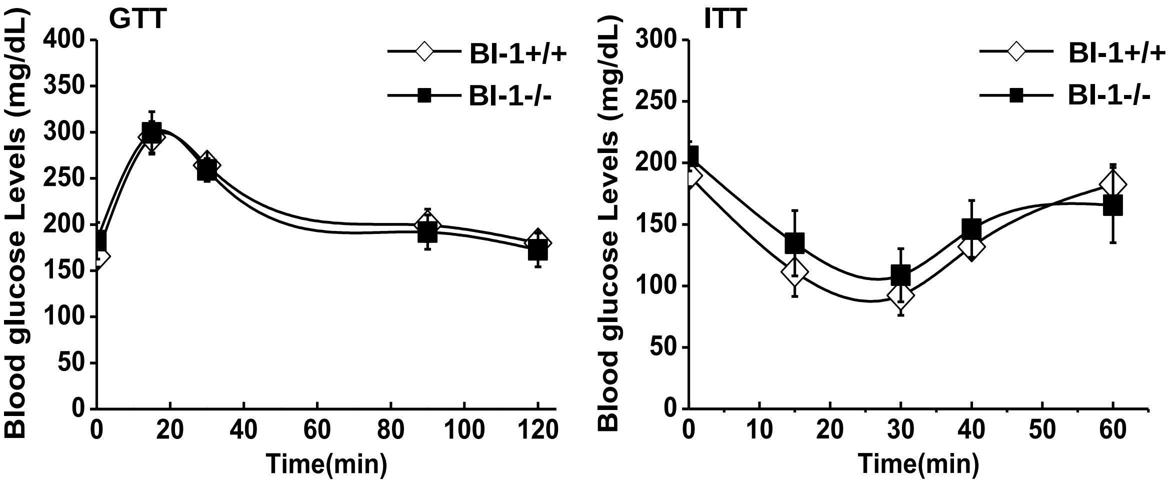
GTT
BI-1+/+
BI-1-/-
Blood glucose Levels (mg/dL)
400
350
300
250
200
150
100
50
0
0
20
40
60
80
100
120
Time(min)
ITT
BI-1+/+
BI-1-/-
Blood glucose Levels (mg/dL)
300
250
200
150
100
50
0
0
10
20
30
40
50
60
Time(min)

Sup Fig 2

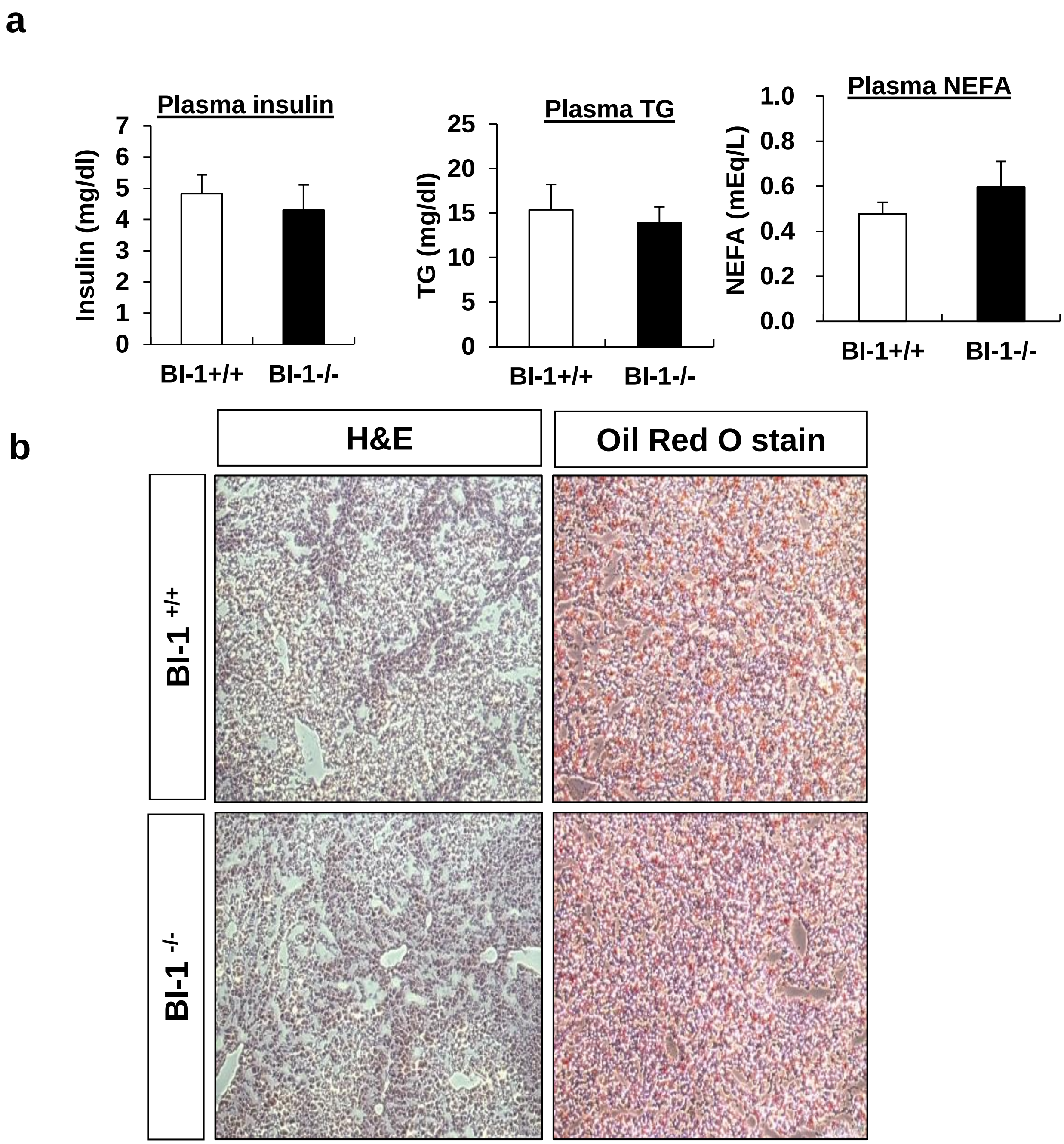
a
Plasma insulin
Insulin (mg/dl)
7
6
5
4
3
2
1
0
BI-1+/+
BI-1-/-
Plasma TG
TG (mg/dl)
25
20
15
10
5
0
BI-1+/+
BI-1-/-
Plasma NEFA
NEFA (mEq/L)
1.0
0.8
0.6
0.4
0.2
0.0
BI-1+/+
BI-1-/-
b
H&E
Oil Red O stain
BI-1 +/+
BI-1 -/-

Sup Fig 3

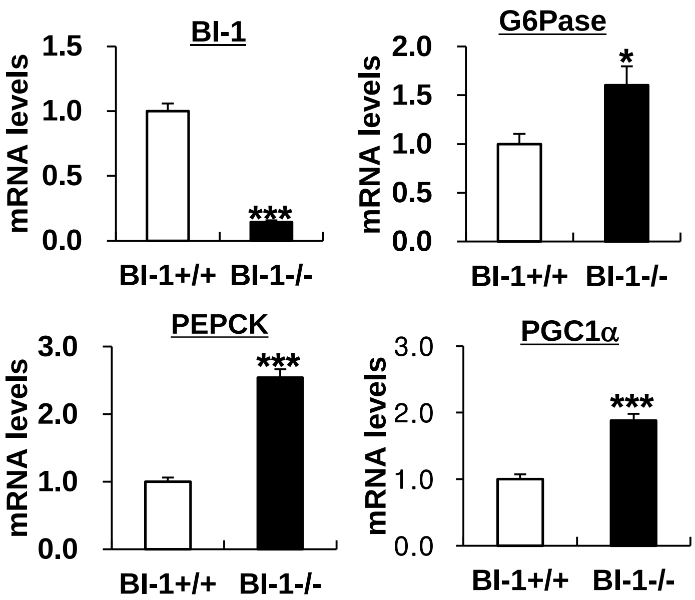
BI-1
mRNA levels
1.5
1.0
0.5
0.0
***
BI-1+/+
BI-1-/-
G6Pase
mRNA levels
2.0
1.5
1.0
0.5
0.0
*
BI-1+/+
BI-1-/-
PEPCK
mRNA levels
3.0
2.0
1.0
0.0
***
BI-1+/+
BI-1-/-
PGC1α
mRNA levels
3.0
2.0
1.0
0.0
***
BI-1+/+
BI-1-/-

Sup Fig 4

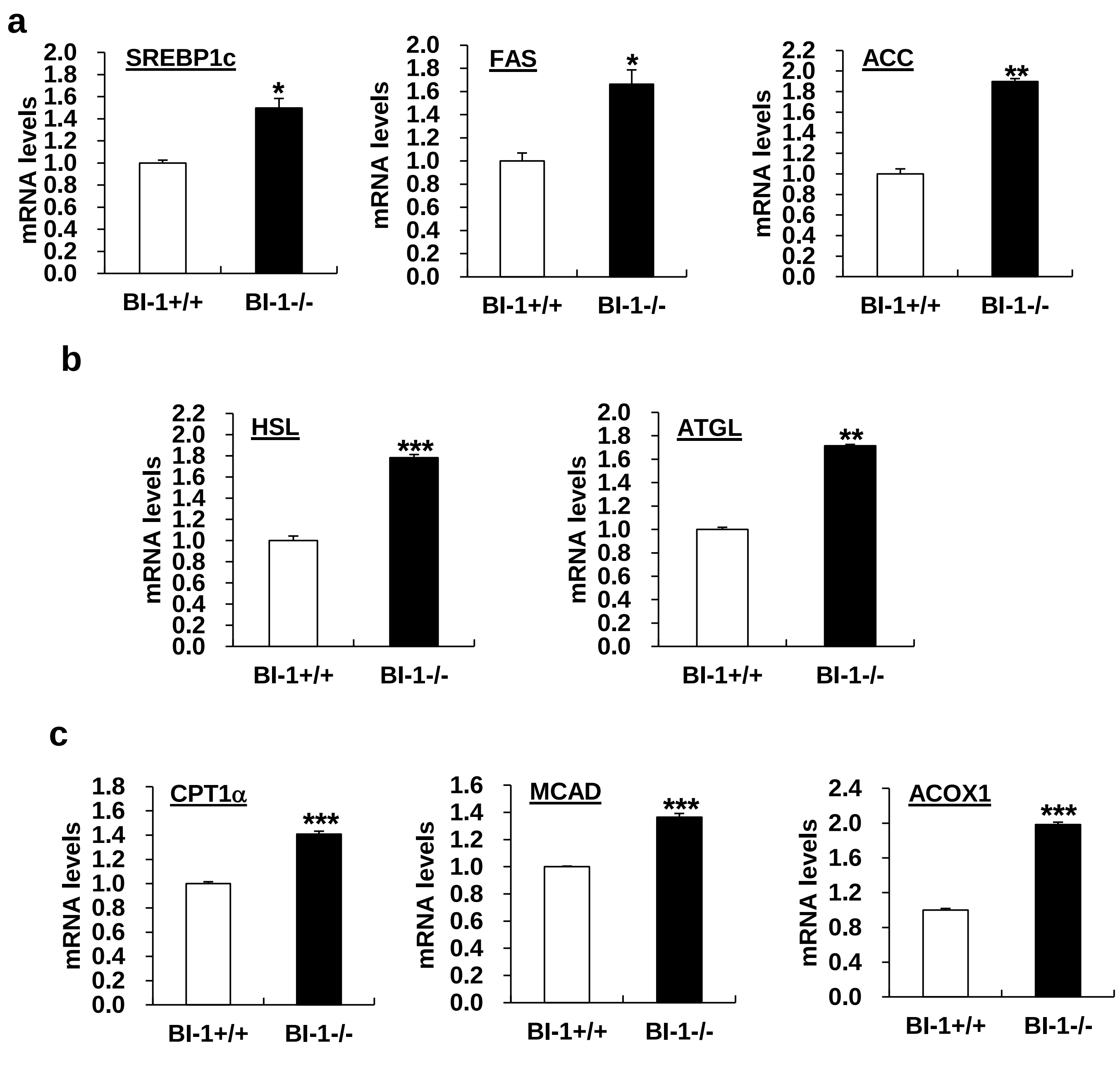
a
SREBP1c
FAS
ACC
mRNA levels
*
**
2.2
2.0
1.8
1.6
1.4
1.2
1.0
0.8
0.6
0.4
0.2
0.0
BI-1+/+
BI-1-/-
b
HSL
ATGL
***
c
CPT1α
MCAD
ACOX1
2.4
1.2
0.8
0.4

Sup Fig 5

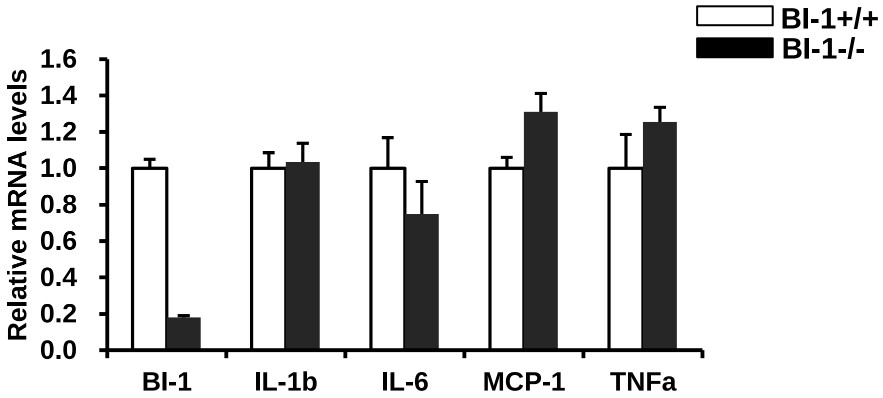

BI-1+/+
BI-1-/-
Relative mRNA levels
1.6
1.4
1.2
1.0
0.8
0.6
0.4
0.2
0.0
BI-1
IL-1b
IL-6
MCP-1
TNFa

Sup Fig 6

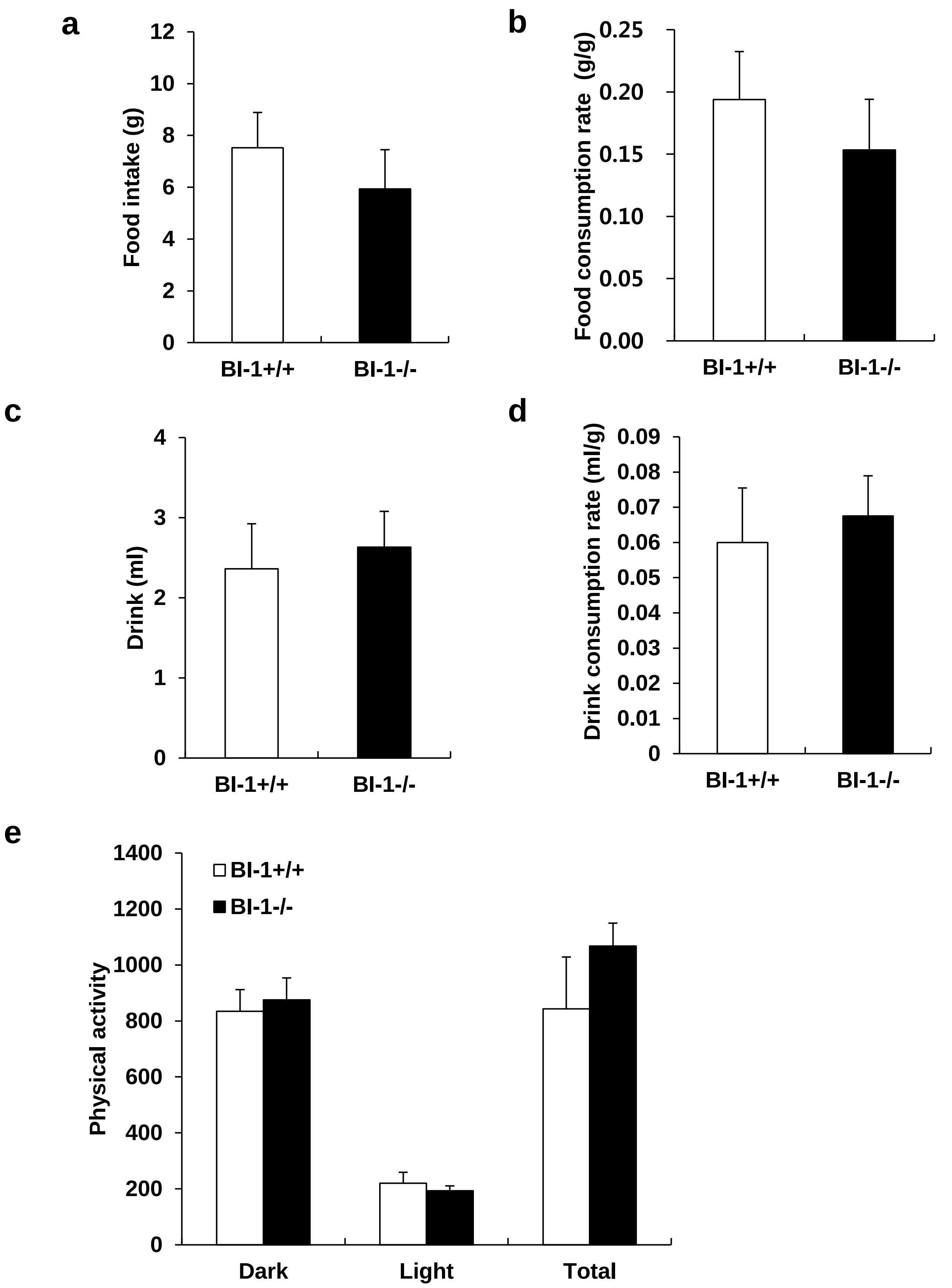

a
12
10
8
6
4
2
0
Food intake (g)
BI-1+/+
BI-1-/-
b
0.25
0.20
0.15
0.10
0.05
0.00
Food consumption rate (g/g)
BI-1+/+
BI-1-/-
c
4
3
2
1
0
Drink (ml)
BI-1+/+
BI-1-/-
d
0.09
0.08
0.07
0.06
0.05
0.04
0.03
0.02
0.01
0
Drink consumption rate (ml/g)
BI-1+/+
BI-1-/-
e
1400
1200
1000
800
600
400
200
0
Physical activity
BI-1+/+
BI-1-/-
Dark
Light
Total

Sup Fig 7

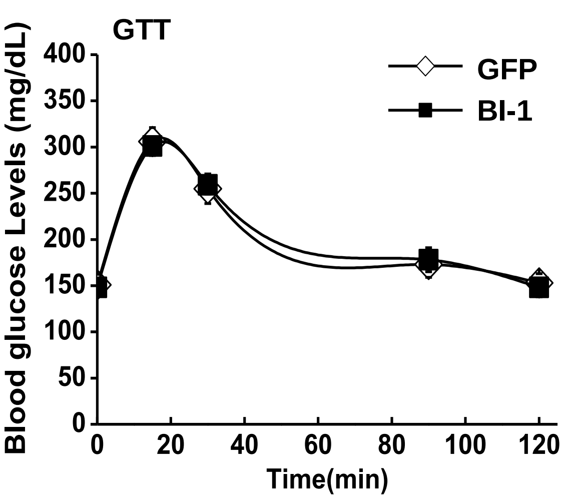
GTT
Blood glucose Levels (mg/dL)
400
350
300
250
200
150
100
50
0
0
20
40
60
80
100
120
Time(min)
GFP
BI-1
ITT
Blood glucose Levels (mg/dL)
300
250
200
150
100
50
0
0
10
20
30
40
50
60
Time(min)
GFP
BI-1

Sup Fig 8

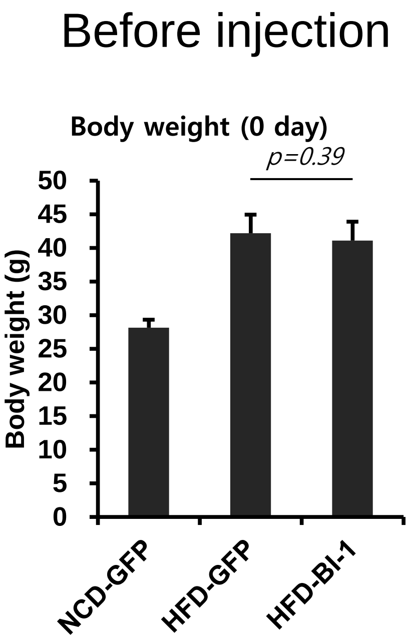

Before injection
Body weight (0 day)
p=0.39
50
45
40
35
30
25
20
15
10
5
0
Body weight (g)
NCD-GFP
HFD-GFP
HFD-BI-1

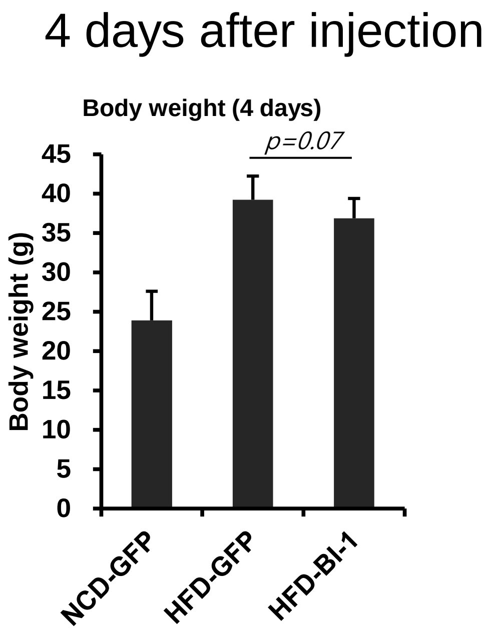

4 days after injection
Body weight (4 days)
p=0.07
45
40
35
30
25
20
15
10
5
0
Body weight (g)
NCD-GFP
HFD-GFP
HFD-BI-1

Sup Fig 9

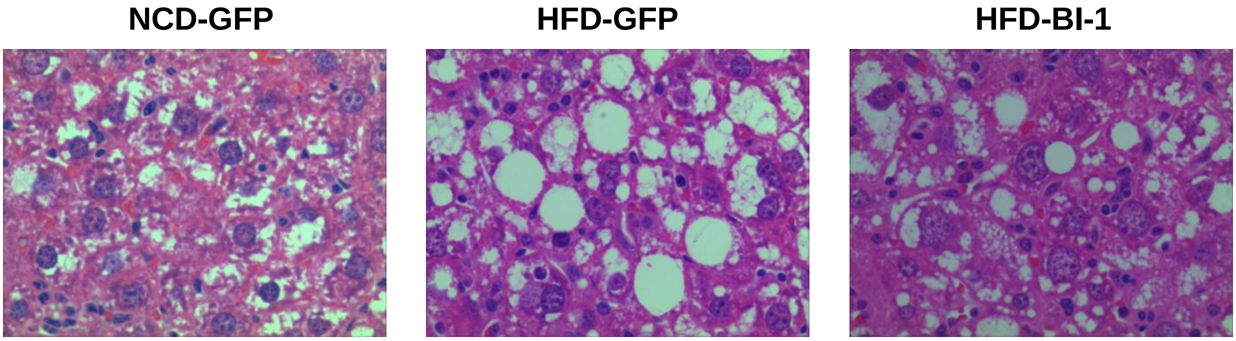

Sup Fig 10

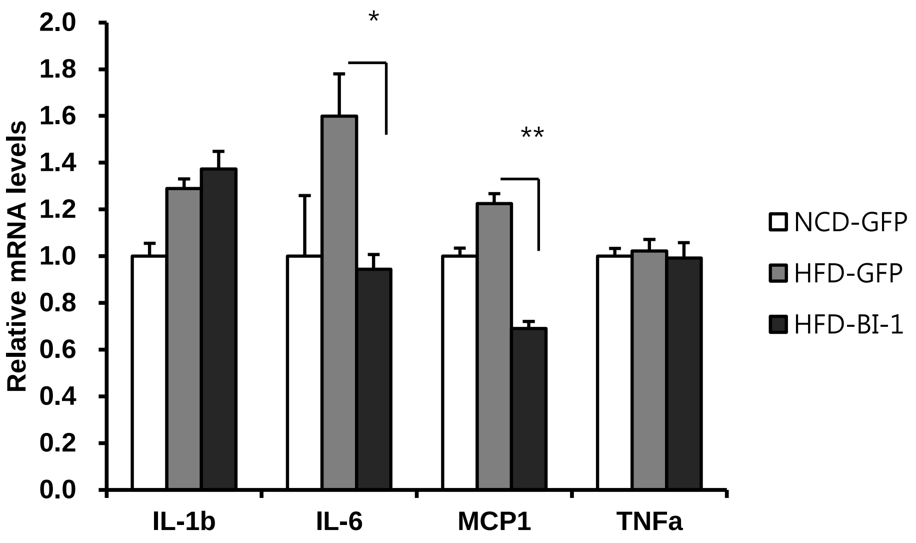

2.0
1.8
1.6
1.4
1.2
1.0
0.8
0.6
0.4
0.2
0.0
Relative mRNA levels
*
**
IL-1b
IL-6
MCP1
TNFa
NCD-GFP
HFD-GFP
HFD-BI-1

Sup Fig 11

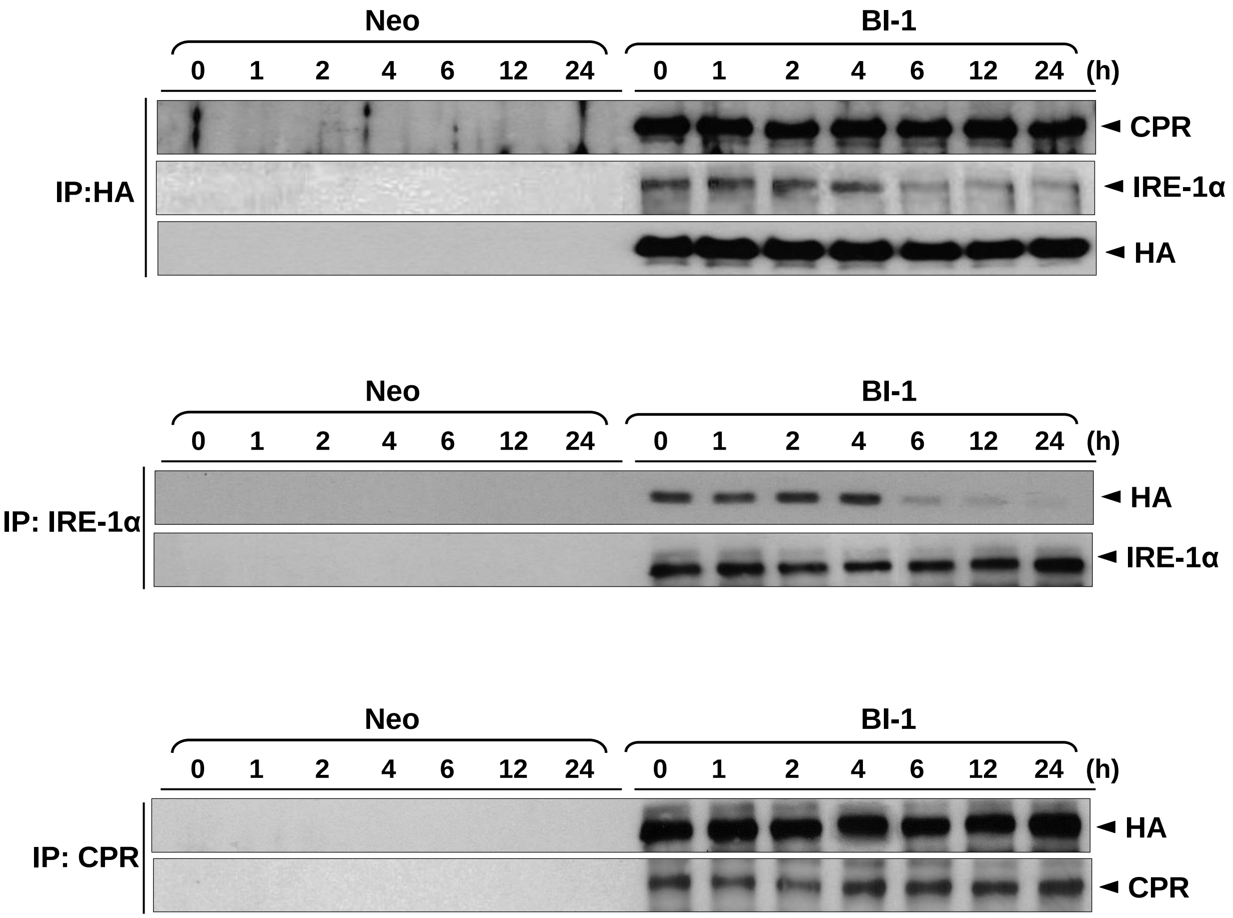
Neo
BI-1
0 1 2 4 6 12 24
0 1 2 4 6 12 24 (h)
IP:HA
CPR
IRE-1α
HA
Neo
BI-1
0 1 2 4 6 12 24
0 1 2 4 6 12 24 (h)
IP: IRE-1α
HA
IRE-1α
Neo
BI-1
0 1 2 4 6 12 24
0 1 2 4 6 12 24 (h)
IP: CPR
HA
CPR
